# Developing Diagnostic Frameworks in Veterinary Behavioral Medicine: Disambiguating Separation Related Problems in Dogs

**DOI:** 10.3389/fvets.2019.00499

**Published:** 2020-01-17

**Authors:** Luciana S. de Assis, Raquel Matos, Thomas W. Pike, Oliver H. P. Burman, Daniel S. Mills

**Affiliations:** ^1^Animal Behaviour, Cognition and Welfare Research Group, School of Life Science, University of Lincoln, Lincoln, United Kingdom; ^2^Faculty of Veterinary Medicine, University Lusófona of Humanities and Technologies, Lisbon, Portugal

**Keywords:** canine, behavioral problems, questionnaire, diagnosis, separation related disorders, separation anxiety, emotion, *Canis familiaris*

## Abstract

Diagnoses are widely used in both human and veterinary medicine to describe the nature of a condition; by contrast, syndromes are collections of signs that consistently occur together to form a characteristic presentation. Treatment of syndromes, due to either their lack of a clear biological cause or multiple causes, necessarily remains non-specific. However, the discovery of interventions may help refine the definition of a syndrome into a diagnosis. Within the field of veterinary behavioral medicine, separation related problems (SRPs) provide a good example of a syndrome. We describe here a comprehensive process to develop a diagnostic framework (including quality control assessments), for disambiguating the signs of SRPs as an example of a heterogeneous behavioral syndrome in non-human animals requiring greater diagnostic and treatment precision. To do this we developed an online questionnaire (243 items) that covered the full spectrum of theoretical bases to the syndrome and undertook a large-scale survey of the presenting signs of dogs with one or more of the signs of SRPs (*n* = 2,757). Principal components analysis (*n*1 = 345), replicated in a second sample (*n*2 = 417; total *n* = 762), was used to define the structure of variation in behavioral presentation, while hierarchical agglomerative cluster analysis cross checked with the partitioned around medoids method was used to determine sub-populations. A total of 54 signs were of value in defining a latent structure consisting of seven principal components (termed “exit frustration,” “social panic,” “elimination,” “redirected frustration,” “reactive communication,” “immediate frustration,” “noise sensitivity”), which divided the population in four clusters (termed “exit frustration,” “redirected reactive,” “reactive inhibited” and “boredom” related SRPs) with 11 sub-clusters (3, 3, 3, and 2, respectively). We used a bottom-up data-driven approach with numerous quality checks for the definition of robust clusters to provide a robust methodology for nosological studies in veterinary behavioral medicine, that can extend our understanding of the nature of problems beyond SRPs. This provides a solid foundation for future work examining aetiological, and differential treatment outcomes, that will allow both more effective treatment and prevention programmes, based on a fully appreciation of the nature of the problem of concern.

## Introduction

Even though both the research base and practice of veterinary behavioral medicine has grown rapidly over the last 20 years, the field still lacks standardized protocols for diagnosis that reliably tease out different psychological forms of common presenting complaints. Thus, there is a danger that a syndrome such as “separation anxiety” is seen as a diagnosis, when the relative significance of emotions such as fear, frustration and the panic associated with loss of an attachment figure may be fundamentally important to understand for effective treatment. In this study we highlight the importance of distinguishing between “diagnoses” and “syndromes” using separation related problems in dogs as an example. We describe and demonstrate a method for identifying meaningful behavioral clusters that are hypothesized to be related to different psychological states that not only form a sound basis to differentials that can be tested scientifically using the hypothetico-deductive method by researchers, but also used by clinicians to enable the implementation of more precise and thus less demanding treatment programs.

### Diagnoses vs. Syndromes

Diagnoses are widely used in both human and veterinary medicine to describe the nature of a condition; by contrast, syndromes in the human medical field are collections of signs that consistently occur together to form a characteristic presentation which initially do not have a known cause (e.g., the initial identification of Down's syndrome by Victorian physician John Langdon Down). In some cases this remains the situation, as is the case with irritable bowel syndrome (1); however, certain conditions with a known cause which might present with similar signs are not included in the syndrome (e.g., celiac disease is not part of irritable bowel syndrome). Within the veterinary disciplines the term is frequently maintained to refer to problems with several identifying features and no clear underlying cause [e.g., headshaking syndrome in horses—(2)]. Description of a complaint in terms of both a syndrome and diagnosis facilitates coherent research into the phenomenon; but specific rational, scientifically based treatment depends on a diagnosis (3). Treatment of syndromes, by virtue of their lack of a clear biological cause, necessarily remains non-specific, although the discovery of specific interventions may help refine the definition of a syndrome into a diagnosis. Within the field of problem animal behavior (veterinary behavioral medicine) separation related problems in dogs provide a good example of a syndrome as well as the problems associated with confusing “a syndrome” with “a diagnosis.”

### Issues With Separation Related Problems in Dogs

The term separation related problems (SRP) is used here to refer to behavior that is problematic for an owner when their dog is left alone, regardless of cause. Between 22.3–55% of the general dog population are believed to show these signs (4–6), and they make up between 14 and 40% of dog behavior referral cases (7–12). Although these cases are relatively easy to identify, there is some debate over the different forms of the problem, and these cases may be variously described as having “separation anxiety,” “separation related disorders” or “separation related problems” (4, 7, 13–21). There is also undoubtedly inconsistency in the use of this terminology (22) since there is no “diagnostic” test that defines a specific underlying biological mechanism. Defining the construct of interest effectively is not a problem unique to SRPs; it has been recognized more widely in the field of abnormal psychology/psychiatry for some time (23) but still remains a challenge [e.g., Alzheimer's dementia—(24)]. Accordingly, it is not surprising that research into SRPs in dogs can result in confusing, inconsistent or even contradictory findings. For instance, Flannigan and Dodman (25) and Storengen et al. (12) report that neutering increases the risk of these problems more than threefold while McGreevy and Masters (26) found that intact dogs were at higher risk. These contradictory results might be due to genuine regional-related differences between populations, however, it is more likely due to different case definitions, highlighting the lack of scientifically defendable criteria for defining the problem.

Research-related definitions of this syndrome often refer to simple collections of signs [e.g., (27, 28)]. These are not precise enough to make consistent inferences about underlying mechanisms, which are important to recognize when it comes to proposing specific treatment for the individual within a clinical setting. It is clear that several possible psychological processes may account for the collection of behavioral signs that make up these definitions of SRPs, such as fear, frustration and the emotional panic associated with separation from an attachment figure (29). Accordingly, although using an imprecise definition may produce statistically significant results at the population level, these have poor specificity with underlying constructs of interest, such as attachment (21, 28). Further evidence in support of the motivational and emotional heterogeneity of separation related problems includes video observations of dogs with these problems when left alone (13, 30). Lund and Jorgensen (13) suggest that in some subjects the changes in behavior over time when left alone are consistent with a shift in their arousal and/or emotional state, e.g., from frustration to increased fear. Accordingly, it is very challenging to integrate the findings from traditional “basic scientific” methodologies that focus on population level differences into a clinical setting focused on the individual (31). This highlights a fundamental difference in the type of scientific knowledge required for good science vs. good clinical practice. “Basic science” typically concerns itself with establishing the general laws and mechanisms underlying a phenomenon (i.e., has a nomothetic approach to epistemology), with hypothesis-testing done at the level of population averages. By contrast, clinical practice, which seeks to use this information practically, often has to focus on understanding as fully as possible the factors relevant to a specific individual (i.e., has an idiographic approach epistemologically) in order to propose precise treatments. Knowing that “on average” something is true about patients with a given complaint, is of limited value when trying to manage the specific patient in front of you. Taxonomic precision is typically more important for clinical success than it is for statistical success.

Within a clinical context, some have tried to improve the precision and characterization of “separation anxiety” by reference to certain necessary and sufficient criteria [such as the inclusion of signs of distress as a necessary feature of the diagnosis of separation anxiety e.g., (32)]. However [as is evident from the ongoing problems of nosology in the field of human psychiatry (33)], this approach does not overcome the problem of the lack of a specific biological diagnosis onto which treatments can be reliably mapped (34, 35). The inability to reliably make precise inferences about these underlying states, means that at a clinical level, many interventions for this problem are often quite extensive, frequently with non-specific elements [e.g., (36, 37)] addressing several potential emotional responses simultaneously (for example the teaching of a “settle” response on a mat away from the owner, might reduce anxiety at separation or increase frustration tolerance). A lack of specificity also makes treatment potentially more laborious for owners, which may reduce compliance and increase the chance of treatment failure (38, 39). A further consequence of poor definition, and arguably of greater concern, is the potential recommendation of contradictory or even contra-indicated interventions without specification as to when one might be indicated over another. An example of the former is the often recounted recommendation to desensitize a dog to predeparture cues whilst also recommending that a “special chew toy, food filled toy” be left with the dog when preparing to depart (40), which can obviously increase departure predictability. Contra-indicated recommendations include the ignoring of contact seeking behaviors (36), ostensibly to reduce a supposed hyper-attachment, but if the behavior is in fact a sign of anxious attachment, such a response from the carer can be predicted to antagonize the situation or create a more insecure attachment (41). Fundamentally, it needs to be recognized that the terms “separation anxiety,” “separation related problems,” and “separation related disorders” are all used to refer to a syndrome that is ambiguously and/or vaguely defined due to a lack of good empirical data. At its most basic level, the syndrome is defined by the co-occurrence of certain behaviors (destructiveness, elimination and/or vocalization) in a given context (the real or virtual absence of the owner) with a certain level of regularity (e.g., occasionally through to every time the animal is left alone).

### Diagnosing Separation Related Problems

To disambiguate any behavioral syndrome and lay the foundation for more precise potential diagnoses, it is necessary to examine the pattern of a wide range of potential signs of value, without imposing any preconceived diagnostic belief. To do this, large data sets are required and multiple checks to reduce the chance of spurious results. The patterning of signs may then be used to create a more precise taxonomy that can facilitate better diagnoses. Diagnostic categories should be logical, scientific hypotheses concerning the proximate psychological mechanisms involved. This means it is necessary to make reference to not only the behaviors of the syndrome and their context, but also their motivational and emotional basis (29). Motivation and emotion can only be inferred from less direct measures and so they remain hypothetical constructs, but they should be amenable to falsification in accordance with the scientific method (42).

Therefore, in this paper, we describe for the first time a comprehensive process to develop a diagnostic framework (including quality control assessments) for separation related problems in dogs that satisfies the dual demands of a nomothetic approach (by describing statistically defined groups sampled from a diverse population) and the idiographic approach (by describing the specific characteristics which may be used to allocate an individual to one of these groups). Our approach starts from an atheoretical perspective, in order to allow groups to emerge without item sample bias (for example if you only ask about attachment then groups will inevitably emerge on the basis of attachment features, regardless of its importance to the condition). Accordingly, no hypotheses concerning groupings and phenotypes of dogs were stated *a priori*, but only that the different behavioral groupings could possibly be related to different psychological (motivational and emotional) processes. To do this we undertook a large-scale survey of the current signs of dogs presenting with one or more of the signs of separation related problems in order to evaluate variation in behavioral presentation. We then examined how these processes tended to cluster together within individuals to form distinct sub-populations. Importantly, at each stage we undertook a number of quality checks to minimize the risk of spurious results.

## Materials and Methods

### Subjects and Questionnaire

Data were collected during January to November of 2014 through an online questionnaire using Survey Monkey^®^. It was advertised utilizing press releases, and social media networks such as animal protection societies, kennel clubs, dog trainers, behavior consultants, veterinarians, scientific societies for the study of animal behavior and Facebook. The stated inclusion criteria for completion of the questionnaire were that the respondent must be the owner for at least 1 month of a dog currently aged 12 weeks or more. Dogs should currently present with at least one of the following behaviors when separated from their owner or left alone: depression/sadness, destructiveness, vocalization (whining, barking, or howling), or house soiling in order to capture a broad definition of separation related problems for at least a month. In order to minimize the risk of bias from recall, owners were asked not to complete the survey if the dog had previously shown these signs but no longer did so, or if there had been a significant change in the household in the last month (e.g., family member left home, moved house) in order to eliminate transient problems. As a further control, in the present study, we analyzed data only from dogs who were aged over 6 months, to minimize the inclusion of puppy related problems such as chewing due to teething (43) and house soiling due to lack of housetraining.

The questionnaire consisted of 228 closed and 15 open questions (243 items in total) divided into three parts: Owner demographic information (features of the owner and household—eight items); Dog demographic information (age, gender, reproductive status, breed, weight, acquisition source and age, place where it spends the day, and health information-−12 items); and Dog behavior & owner measures associated with separation periods (223 items—see Supplementary Material). The latter referred to both general and detailed information concerning the frequency, intensity and context of the behaviors performed by dogs during owner absence (destructiveness, vocalization, house soiling, and depression/sadness) and their impact on the owner's life. In addition, it included questions about the dog's interactions with them in the home, pre- and post-departure routine and reaction, as well as signs of anxiety, noise sensitivity, frustration, and aggressive behavior drawn from a review of the literature and one of the author's (DM) experience as a veterinary clinical behaviorist of more than 25 years.

Where relevant, items had the option of a “Do not know” response, as it was recognized that many owners may be unsure about their dog's behavior in certain contexts and we wanted to encourage honest reporting, as far as possible (20).

### Data Analysis

Since our aim was to identify behavioral patterns relating to the syndrome of separation related problems, 157 variables related exclusively to the behavior of subjects were assessed, i.e., historic or demographic information were not included in the analysis. In order to maximize the integrity of the data and avoid risks from imputation, any questionnaires with at least one “Do not know” response were initially rejected from analysis.

The preparation (e.g., cleaning, coding, transformation of variables, excluding subjects) of data was carried out using Excel 2010. Since some items were categorical, and to ensure that variation in the range of scores possible for a given item did not skew its potential contribution to the total variance, all item scores were transformed into binary data (44, 45). This meant 29 ordinal items had their frequency scores collapsed into the binary outcomes “never/rare/sometimes” vs. “often/always” (for 10 questions that had three levels of frequency instead of five, the dichotomy was made between “never/sometimes” vs. “always”), with 21 nominal items transformed into binary dummy variables (presence or absence of each element within it).

#### Statistical Analysis

##### Principal component analysis

The statistical analysis was undertaken using R 3.5.1 (46). All 157 variables related to behavior were included in an initial Principal Component Analysis (PCA) in order to identify the main behaviors contributing to the variance in the sample and their correlations with each other. It was conducted in 345 dogs using the “psych” package [function “principal”—(47)] with an oblique rotation [package “GPArotation,” function “oblimin”—(48)] since it was assumed that the variables could potentially correlate among themselves, given that behaviors are not necessarily uniquely associated with a single motivational or emotional state. The number of components to extract was determined from the point of inflection of the scree plot alongside the Kaiser criterion [i.e., PC's must have an Eigenvalue >1—(49–51)].

For interpretative purposes, only items with loadings above 0.4 were retained and considered (52). The resulting principal components (PCs) were presented to a group of five clinical behavior experts within the Animal Behavioral Clinic of the University of Lincoln, in order to develop a relevant consensus interpretation of their emotional and/or motivational content. All experts received a document containing the behaviors grouped into their principal components from the PCA. They were asked what clinical meaning, if any they might attach to these groupings and to interpret, as far as possible, in terms of possible underlying emotion, motivation or severity. All clinicians were familiar with the approach described in Mills (42) for distinguishing motivation (behavioral goal) from emotion (personal functional relationship with stimulus) and triangulating evidence in relation to four components of emotion in order to make a diagnostic assessment on the basis of the scientific principle of falsification.

When this process had been completed, we returned to the dataset to identify subjects who had been excluded initially due to uncertain responses (i.e., “I don't know”) in any of the 157 variables, but who now had complete data for the retained items (56 variables). This resulted in sufficient subjects for the PCA process to be repeated with a second dataset (*n* = 417 dogs) as a form of confirmatory analysis of the robustness of the initial structure identified.

**Figure 1:**
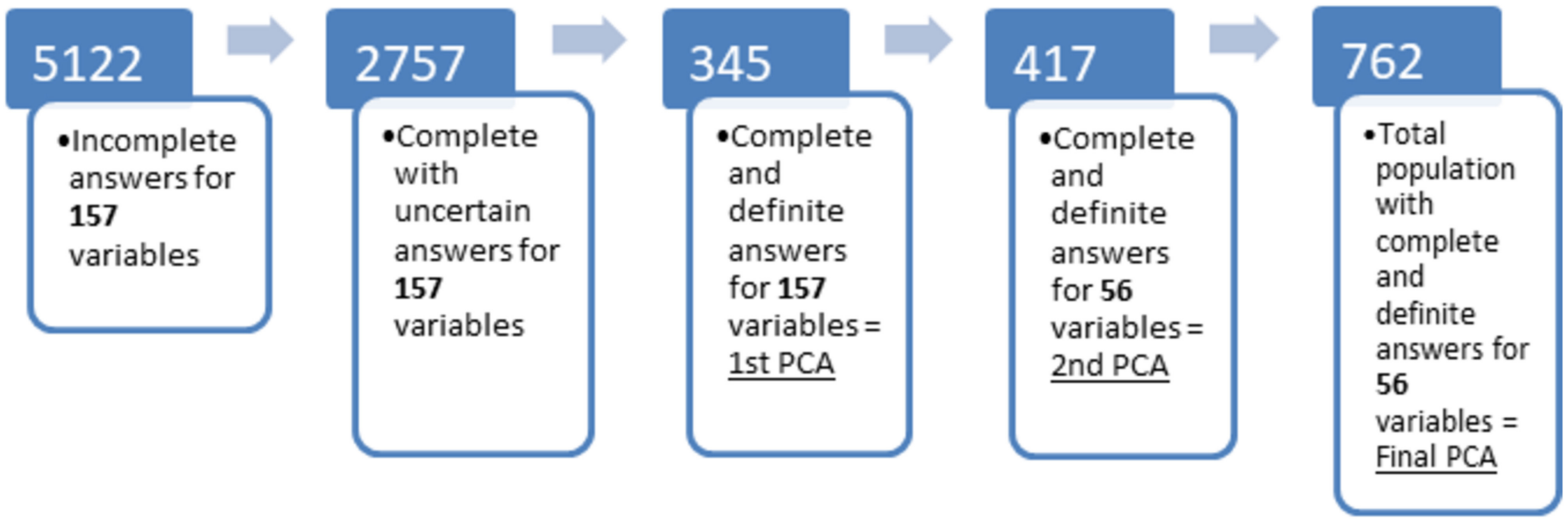
Flow diagram showing population at different stages of analysis of the original survey. (1) total number of respondents including incomplete questionnaires; (2) number of questionnaires that were complete but had at least one uncertain response, i.e., “I don't know”; (3) number of questionnaires that were complete without any uncertain responses for any of the 157 variables of interest; (4) number of questionnaires that were complete and had definite responses for the 56 variables of interest which loaded on the components identified by the first PCA, but which had not been included in stage 3 due to uncertain answers in some of the other 101 items; (5) total number of questionnaires with complete and definite answers for the 56 behavioral variables identified at stage 3. Principal component analysis was performed on the populations represented by the last 3 stages.

The same statistical process (i.e., PCA) was then repeated (i.e., third time) using all subjects used in the previous two analyses as a single population (*n* = 762 dogs, henceforth referred to as the complete data set) in order to produce and interpret a final PCA, as shown in Figure 1.

##### Cluster analysis

In order to determine how the population was structured with respect to the variables identified by the PCA, cluster analysis was used. Cluster analysis involves grouping items on the basis of some statistical measure of similarity, with different methods of cluster analysis using different metrics. To do this a PC score was calculated for each dog in the complete data set based on their owners' response to all relevant items retained within each principal component divided by the number of items making up that component. Items were not weighted according to their loading, since from a clinical perspective, all items were now binary and so their occurrence or not within a PC was the only measure of interest. By dividing each PC by the number of items, each PC had the same standardized score range (0 to +1) regardless of the number of items making it up. This ensured each PC was given equal weighting in the next phase of analysis.

The standardized scores for each PC were then used to perform an exploratory Hierarchical Agglomerative Cluster Analysis (HACA) using Euclidean distance and Ward linkage [“cluster” package, function “agnes”—(53)] in order to group the dogs into different clusters according to their similarity of these signs. The resulting dendrogram was then evaluated and a cut-off point defined at the lowest possible height that maintained the highest degree of separation between clusters and the highest number of viable clusters. In order to validate the robustness of the number of clusters extracted, a second method of cluster analysis was used based on the partitioned around medoids method [CAPAM—e.g., (54); package “cluster,” function “pam”—(53)] using the same number of clusters as had been identified using the HACA. This method of comparing two different clustering methodologies is used in human medical research with similar objectives, i.e., to identify different presentations of a disease or condition [e.g., (55)]. The number and features of each group obtained by the two methods are then compared alongside the percentage agreement between the two methods in their assignment of subjects to the groups.

##### Comparing groups

For each group identified using the HACA method, the average score of each PC was calculated. Data within groups did not show multivariate normality [package “mvnormtest,” function “mshapiro.test”—(56)]. In order to identify which PCs created the differences between clusters a Kruskal-Wallis test was used [package “stats,” function “kruskal.test,” or “wilcox.test”—(57)] with a Dunn test used for *post-hoc* comparisons [package “FSA,” function “dunnTest”—(58, 59)]. Finally, False Discovery Rate Analysis was used to correct for multiple comparisons [package “fdrtool,” function “fdrtool”—(60)].

##### Discriminant analysis

A flexible discriminant analysis was then used to determine how effectively subjects within the clusters could be distinguished using their PC scores and if so, how to effectively do it in new individuals. This method was chosen as it allows for the analysis of non-parametric data by using function “fda” from package “mda” (61).

This process was then repeated for each cluster in order to evaluate whether each main cluster could be meaningfully divided into sub-clusters which might represent important clinical sub-populations within a cluster.

## Results

From an initial sample of 5,122 responses (collected during January to November of 2014), 2,839 were fully completed. After exclusion of dogs aged 6 months or less, 2,757 dogs remained in the dataset (Figure 1).

When those with at least one uncertain response (i.e., “I don't know”) for any one of the 157 variables were excluded, 345 (Figure 1) subjects remained with complete known data for the initial PCA [a minimum of 100 is recommended to reduce the risk of mathematical artifacts on the results (62)]. With 157 variables of interest, the ratio of subjects to items exceeded the recommended minimum threshold of 2:1 for principal components analysis (62). Using the point of inflection of the scree plot and Kaiser criterion, seven principal components (PCs) were considered with 56 items loading >0.4 on these 7 PCs retained [(63), see Supplementary Material].

Upon returning to the data set, a further 417 responses were found which had complete known (i.e., without “I don't know”) answers to these 56 variables but had been previously excluded as there were unknown responses to the other 101 items. A second PCA was performed on this subpopulation and showed a similar structure (see Supplementary Material). By summing these two sub-populations the complete dataset of 762 individuals resembled the initial sample (2,757 dogs) as shown in Table 1 and was used in the subsequent analyses (Figure 1). In total 116 breeds were represented in addition to the mixed breeds or unknown breeds which represented most subjects (Table 1).

**Table 1:** Demographic data from two populations of dogs aged over 6 months old for which owners completed the separation related problems questionnaire: completed answers (2,757 dogs) and answers without unsure response, i.e., I don't know (762 dogs).

		**Completed questionnaire for 157 variables**		**Completed questionnaires with definite responses for 56 variables**	
		***N* = 2,757**	**%**	***N* = 762**	**%**
Age	Average (years old)	4.8		5	
	Standard deviation (years)	3.4		3.2	
Gender	Females	1,205	43.7	310	40.7
	Females neutered	984	81.7	256	82.6
	Males	1,552	56.3	452	59.3
	Males neutered	1,192	76.8	342	75.7
Breeds	Mixed breeds/unknown breed	761	27.6	227	29.8
	Weimaraners	106	3.9	32	4.2
	Labrador retrievers	105	3.8	29	3.8
	German shepherd dogs	97	3.5	27	3.5
	Whippets	76	2.8	19	2.5
	Beagles	67	2.4	17	2.2
	Poodles	61	2.2	16	2.1
	Border collies	57	2.1	16	2.1
	Boston terriers	56	2.0	8	1.1
	Boxers	55	2.0	11	1.4
Number of dogs	Lived without another dog	1,232	44.7	296	38.9
	Lived with one dog	904	32.8	262	34.4
	With 2 dogs	348	12.6	112	14.7
	With 3 dogs	130	4.7	90	11.8
	Lived with 4 or more dogs (up to 19 dogs)	143	5.2	2	0.3
Source of acquisition	Purchased from a breeder	1,026	37.2	282	37
	Adopted from a shelter or rescue group	947	34.3	268	35.2
	From neighbors/family	369	13.4	101	13.3
	From street	112	4.1	38	5
	From pet-shop	61	2.2	16	2.1
	Other sources	186	6.8	38	5

Table 2 shows the frequency of each behavior (destructiveness, vocalization (whining, barking, or howling), house soiling and depression/sadness) when separated from their owner or left alone of the 762 dogs behaviors.

**Table 2:** Frequencies of each of the main behaviors performed alone or in combination with others of 762 dogs presenting separation related problems.

	**Signs from the first column**	**Combination of signs: first plus third, fourth, fifth or sixth columns**			
	**Total**	**Vocalization**	**Urination**	**Defecation**	**Depression/sadness**
Destruction	346 (45.4%)	277 (36.4%)	97 (12.7%)	72 (9.5%)	190 (24.9%)
Frequency: usually or always	133 (38.3%)				
Vocalization	591 (77.6%)		171 (22.4%)	119 (15.6%)	321 (42.1%)
Frequency: usually or always	444 (75.1%)				
Urination	213 (28%)			122 (16%)	118 (15.5%)
Frequency: usually or always	67 (31.5%)				
Defecation	150 (19.7%)				83 (10.9%)
Frequency: usually or always	50 (33.3%)				
Depression/sadness	403 (52.9%)				
Frequency: usually or always	118 (29.3%)				
Destruction and vocalization			81 (10.6%)	59 (7.7%)	
House soiling		98 (12.9%)			70 (9.2%)
House soiling and destruction	60 (7.9%)	49 (6.4%)			34 (4.5%)
House soiling, destruction and vocalization					29 (3.8%)

### Behavior Signs Consistently Contributing to the Variation Seen in the Presentation of Separation Related Problems

The PCA on this population of 762 dogs confirmed the seven principal component structure but eliminated two behaviors (i.e., “bark when confined” and “destruction of carpet”), with the new components accounting for 52% of the total variance (See Supplementary Material). The maximum correlation between the principal components was between PC1 and PC4 at 0.37 which exceeds the recommended threshold of 0.32, indicative of the correct selection of an oblique rotation (52).

The first PC was composed of 10 behaviors related to elements of destruction of exit points and explained 11% of total variance; this collection of behaviors was interpreted by the expert panel as reflecting “*exit frustration*.” The 15 behaviors of PC2 were responsible for 9% of total variance and focused on aspects of vocalization and distress, occurring around the time of departure, and so were labeled as “*social panic*” [sensu Panksepp (64)]. The third principal component had 10 behaviors, explained 9% of the variance and was related to house soiling and so was labeled “*elimination*.” PC4 explained 8% of total variance and was composed of six behaviors concerning oral destructiveness, which seems to reflect “*redirected frustration*” in the context of isolation. PC5 was composed of six behaviors related to barking vs. tail wagging (negative loadings) in relation to a range of uncertain social encounters; it was responsible for 6% of the total variance and was labeled “*reactive communication*.” PC6 explained 5% of the variance and its three behaviors related to aggressive behaviors when usual expectations are curtailed or denied, and so may be considered a response to “*immediate frustration*.” Finally, another 5% of total variance was explained by PC7 which was composed of four behaviors related to “panicking” and destruction in response to loud noises, and so was described as “*noise sensitivity*” (Table 3 and Supplementary Material). From here onwards these labels (in italics above) will be used for simplicity to refer to the specific PCs. Further justification for the interpretation is given in the discussion.

**Table 3:** Behaviors of each principal component according to the results of the principal component analysis of 54 behaviors of 762 dogs presenting separation related problems.

**Principal component and interpretation**	**Behaviors**	***N (%)***
PC1—Exit frustration	Destruction of the main exit door when it was closed	121 (15.9)
	Destruction of the main exit door of the room	112 (14.7)
	Destruction of door frame next to where the door opens	93 (12.2)
	Destruction of door itself next to where it opens	87 (11.4)
	Destruction of doors	147 (19.3)
	Destruction on or around door handle	52 (6.8)
	Destruction of floor nearby the place where the door opens	50 (6.6)
	Destruction of big objects (furniture, windows, doors, doorframes, other exit points from house)	197 (25.9)
	Destruction of house structure (holes in wall, torn up linoleum)	110 (14.4)
	Destruction using his/her claws	256 (33.6)
PC2—Social panic	Vocalization after owner has stepped outside	514 (67.5)
	Whines during routinely pre-departures	280 (36.8)
	Whines during unusual pre-departures	319 (41.9)
	Frequency of vocalization when dog is left alone for at least 1 h (often and always)	444 (59.3)
	Paces during routinely pre-departures	305 (40)
	Frequency of whining without human company (always)	254 (33.3)
	Vocalizes without human company	591 (77.6)
	Frequency of distress pre-departure (often and always)	258 (33.9)
	Paces during unusual pre-departures	351 (46.1)
	Frequency of vocalization when dog is left confined for at least 1 h (often and always)	287 (37.7)
	Vocalizes during short separation period	264 (34.7)
	Frequency of restlessness, agitation or pacing when confined of left home alone (often and always)	254 (33.3)
	Looks anxious during short separation period	209 (27.4)
	Bites and/or claws the door/window/crate after owner has stepped outside	231 (30.3)
	Frequency of barking without human company (always)	243 (31.9)
PC3—Elimination	Urinates in inappropriate places in owner absence	213 (27.9)
	Defecates in inappropriate places in owner absence	150 (19.7)
	Urinates in inappropriate places when alone or confined	163 (21.4)
	Defecates in inappropriate places when alone or confined	125 (16.4)
	Urine when alone that started only after 6 months old	238 (31.2)
	Defecates when alone that started only after 6 months old	192 (25.2)
	Frequency of house soiling when left alone for at least 1 h (often and always)	69 (9.1)
	Frequency of house soiling when alone that happened only after 6 months old (often and always)	50 (6.6)
	Urinates in inappropriate places even when not alone or confined	66 (8.7)
	Defecates in inappropriate places even when not alone or confined	43 (5.6)
PC4—Redirected frustration	Takes objects and destroys them when alone without human company	323 (42.4)
	Destruction using his/her mouth	441 (57.9)
	Destruction of medium-sized items	278 (36.5)
	Destruction of clothing	214 (28.1)
	Take objects and destroy them when confined without human company	197 (25.9
	Destructiveness in owner absence	346 (45.4)
PC5—Reactive communication	Barks when there's a person at the door	574 (75.3)
	Wags tail when there's a person at the door^*^	148 (19.4)
	Barks when doorbell rings	582 (76.4)
	Wags tail when he/she is inside of the car and an unfamiliar person/dog approaches^*^	182 (23.9)
	Barks when he/she is inside of the car and an unfamiliar person/dog approaches	388 (50.9)
	Barks when it can't reach an unfamiliar person/dog when approaching	482 (63.3)
PC6—Immediate frustration	Bites when the owner tries to put him/her on the lead earlier than normal after a run in the park or when he/she gets a significantly shorter walk than usual.	2 (0.3)
	Bites when he/she is not allowed to play free in the park or do some other usual activity 1 day	2 (0.3)
	Growls when he/she sees the owner outside talking to some person	1 (0.1)
PC7—Noise sensitivity	Panics and starts to destroy things when hears screeches or whistles	3 (0.4)
	Panics and starts to destroy things when hears fireworks bangers	10 (1.3)
	Panics and starts to destroy things when hears thunderstorms	6 (0.8)
	Panics and starts to destroy things when hears sudden loud noises (e.g., car backfires, objects falling)	5 (0.7)

* *Behaviors that loaded negatively within their principal components (i.e., two items in PC5)*.

### Main Behavioral Profiles Among Dogs With Separation Related Problems

**Figure 2:**
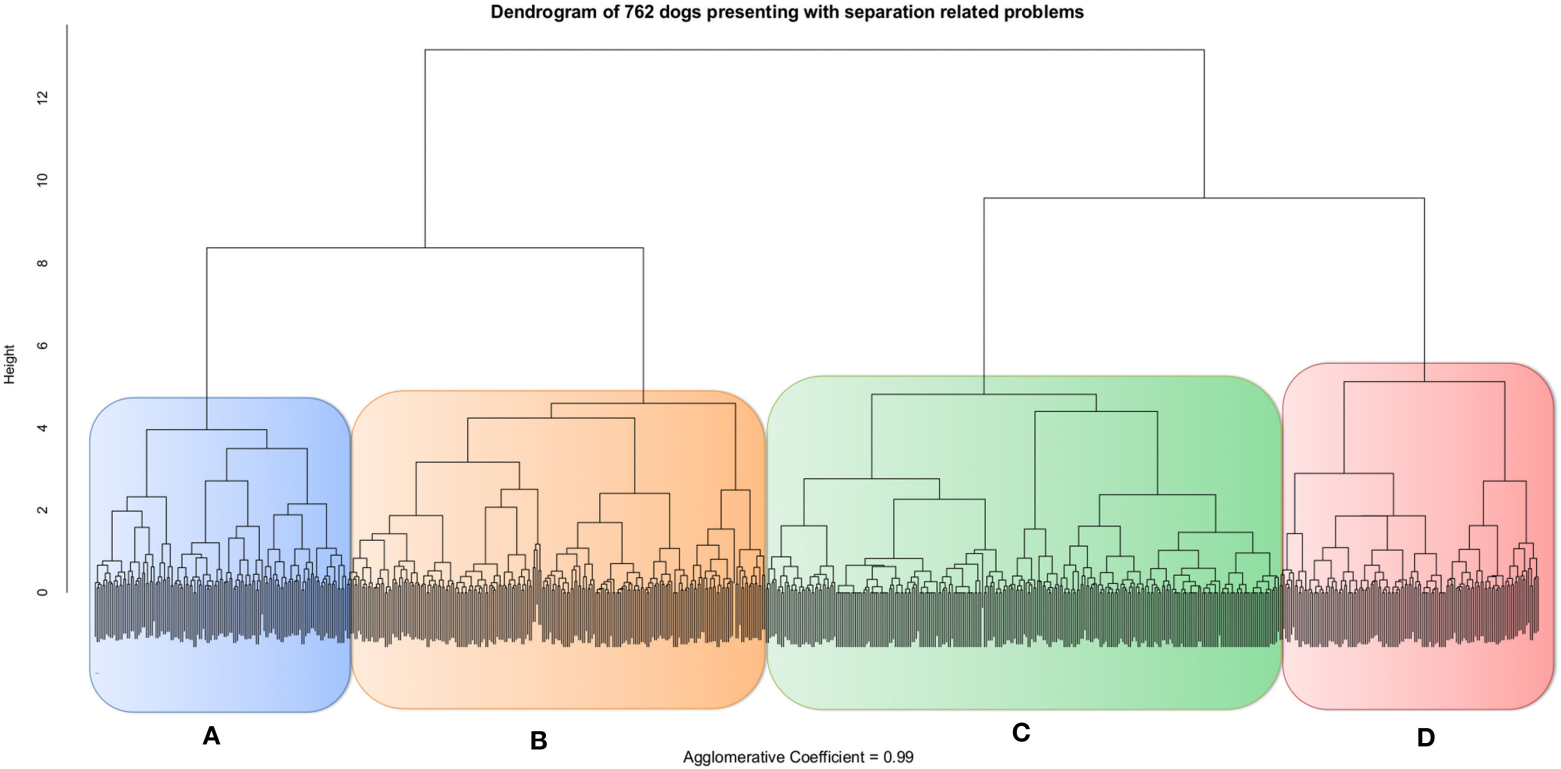
Dendrogram resulting from the hierarchical agglomerative cluster analysis of 762 dogs presenting with separation related problems based on their principal component scores: Exit frustration, Social panic, Elimination, Redirected frustration, Reactive communication, Immediate frustration and Noise sensitivity. Groups **A** (blue), **B** (orange), **C** (green) and **D** (red).

Each dog had a principal component (PC) score calculated by summing all signs scored as ±1 according to their loading on the PC, and then dividing the total by the maximum total of possible signs for each PC. Because “*Reactive communication*” was the only PC with negative items its range of signs was −2 to +4, totalizing six possible results which was also turned into 0–1. In this way seven standardized PC scores were calculated for each dog. These values were then used for the hierarchical agglomerative cluster analysis which indicated subjects grouped into four distinct clusters (Figure 2 and Table 4).

**Table 4:** Number and percentage of 762 dogs presenting with separation related problems assigned to each group according to the type of cluster analysis.

**HACA**	**A (*n* = 133)**	**%**	**B (*n* = 221)**	**%**	**C (*n* = 271)**	**%**	**D (*n* = 137)**	**%**
**CAPAM**								
**E (*****n*** **=** **118)**	***114***	*85*.714	3	1.358	0	0	1	0.73
**%**	*96*.610		2.542		0		0.848	
**F (*****n*** **=** **244)**	17	12.782	***200***	*90*.498	6	2.214	21	15.329
**%**	6.967		*81*.967		2.459		8.607	
**G (*****n*** **=** **118)**	3	2.256	0	0	13	4.797	***104***	75.912
**%**	2.542		0		11.017		*88*.136	
**H (*****n*** **=** **282)**	1	0.752	18	8.145	***252***	*92*.989	11	8.029
**%**	0.355		6.383		*89*.362		3.901	

*Bold and italic numbers indicate to which group most dogs were assigned. Underlined numbers on the right of the bold and italic ones are related to the Hierarchical agglomerative cluster analysis (HACA) while those under the bold and italic are related to the Cluster analysis using partitioning around medoids method (CAPAM)*.

*HACA, hierarchical agglomerative (clusters named A, B, C, and D); and CAPAM, partitioning around medoids (clusters named E, F, G, and H)*.

Eighty-nine percentage of dogs assigned to these four clusters were assigned to the equivalent four clusters specified in the second cluster analysis using CAPAM indicating robustness to the structure (Table 4), with between 93 and 76% agreement for any given cluster.

Four of the PC scores were significantly different between the groups Exit Frustration (*X*^2^: 505.84, df = 3, *p* < 2.2e-16), Social Panic (*X*^2^: 73.352, df = 3, *p* = 8.172e-16), Redirected frustration (*X*^2^ = 502.1, df = 3, *p* < 2.2e-16), and Reactive communication (*X*^2^ = 329.16, df = 3, *p* < 2.2e-16) (See Table 5 for *post hoc* comparisons results). For each PC making up the clusters two average scores were calculated: “*N*_t_” (the average score based on all subjects in that cluster) and “*N*_s_” (the average score for subjects who showed at least one of the signs in that cluster for a given principal component). *N*_t_ represents the typical nomothetic value, being based on all members of the cluster and so is the value used in statistical analysis. By contrast, Ns is the more useful value to a clinician (who is focused on idiographic information), as it indicates the average value given the condition that the dog shows this collection of signs i.e., the mean severity of the PC when it does occur.

**Table 5:** Average score followed by standard deviation of each group according to each principal component of 762 dogs presenting with separation related problems after hierarchical agglomerative analysis.

**Principal components**	***N***	**A (*N*_**t**_ = 133)**	**B (*N*_**t**_ = 221)**	**C (*N*_**t**_ = 271)**	**D (*N*_**t**_ = 137)**
**Exit frustration**	*N*_t_	0.712 ± 0.183^***^^a^	0.096 ± 0.119^***^^b^	0.006 ± 0.033^***^^c^	0.037 ± 0.078^**^^d^
	*N*_s_	0.712 ± 0.183 (*N*_s_ = 133)	0.132 ± 0.121 (*N*_s_ = 160)	0.155 ± 0.069 (*N*_s_ = 11)	0.152 ± 0.087 (*N*_s_ = 33)
**Social panic**	*N*_t_	0.582 ± 0.256^***^^a^	0.408 ± 0.269^***^^b^	0.414 ± 0.270^***^^b^	0.295 ± 0.234^***^^c^
	*N*_s_	0.582 ± 0.256 (*N*_s_ = 133)	0.432 ± 0.258 (*N*_s_ = 209)	0.454 ± 0.249 (*N*_s_ = 247)	0.375 ± 0.199 (*N*_s_ = 108)
**Elimination**	*N*_t_	0.197 ± 0.271	0.174 ± 0.263	0.172 ± 0.243	0.142 ± 0.239
	*N*_s_	0.423 ± 0.249 (*N*_s_ = 62)	0.428 ± 0.246 (*N*_s_ = 90)	0.392 ± 0.22 (*N*_s_ = 119)	0.454 ± 0.199 (*N*_s_ = 43)
**Redirected frustration**	*N*_t_	0.698 ± 0.258^***^^a^	0.701 ± 0.215^***^^a^	0.043 ± 0.87^***^^b^	0.294 ± 0.33^***^^c^
	*N*_s_	0.698 ± 0.258 (*N*_s_ = 133)	0.701 ± 0.215 (*N*_s_ = 221)	0.198 ± 0.066 (*N*_s_ = 59)	0.511 ± 0.279 (*N*_s_ = 79)
**Reactive communication**	*N*_t_	0.723 ± 0.294^**^^a^	0.827 ± 0.197^***^^b^	0.854 ± 0.167^***^^b^	0.202 ± 0.181^***^^c^
	*N*_s_	0.751 ± 0.262 (*N*_s_ = 128)	0.827 ± 0.197 (*N*_s_ = 221)	0.854 ± 0.167 (*N*_s_ = 271)	0.301 ± 0.136 (*N*_s_ = 92)
**Immediate frustration**	*N*_t_	0 ± 0	0.005 ± 0.067	0.003 ± 0.041	0 ± 0
	*N*_s_	0 ± 0 (*N*_s_ = 0)	1 ± 0.0 (*N*_s_ = 1)	0.667 ± 0.0 (*N*_s_ = 1)	0 ± 0 (*N*_s_ = 0)
**Noise sensitivity**	*N*_t_	0.004 ± 0.031	0.020 ± 0.117	0.002 ± 0.021	0.004 ± 0.030
	*N*_s_	0.25 ± 0.0 (*N*_s_ = 2)	0.563 ± 0.291 (*N*_s_ = 8)	0.25 ± 0.0 (*N*_s_ = 2)	0.25 ± 0.0 (*N*_s_ = 2)

*N_t_, all subjects in that cluster; N_s_, subjects showing at least one of the signs in that cluster for a given principal component. Bold PCs are significantly different between clusters, with different superscript letters identifying the source of difference*,

** p <0.01;

*** *p <0.001*.

**Figure 3:**
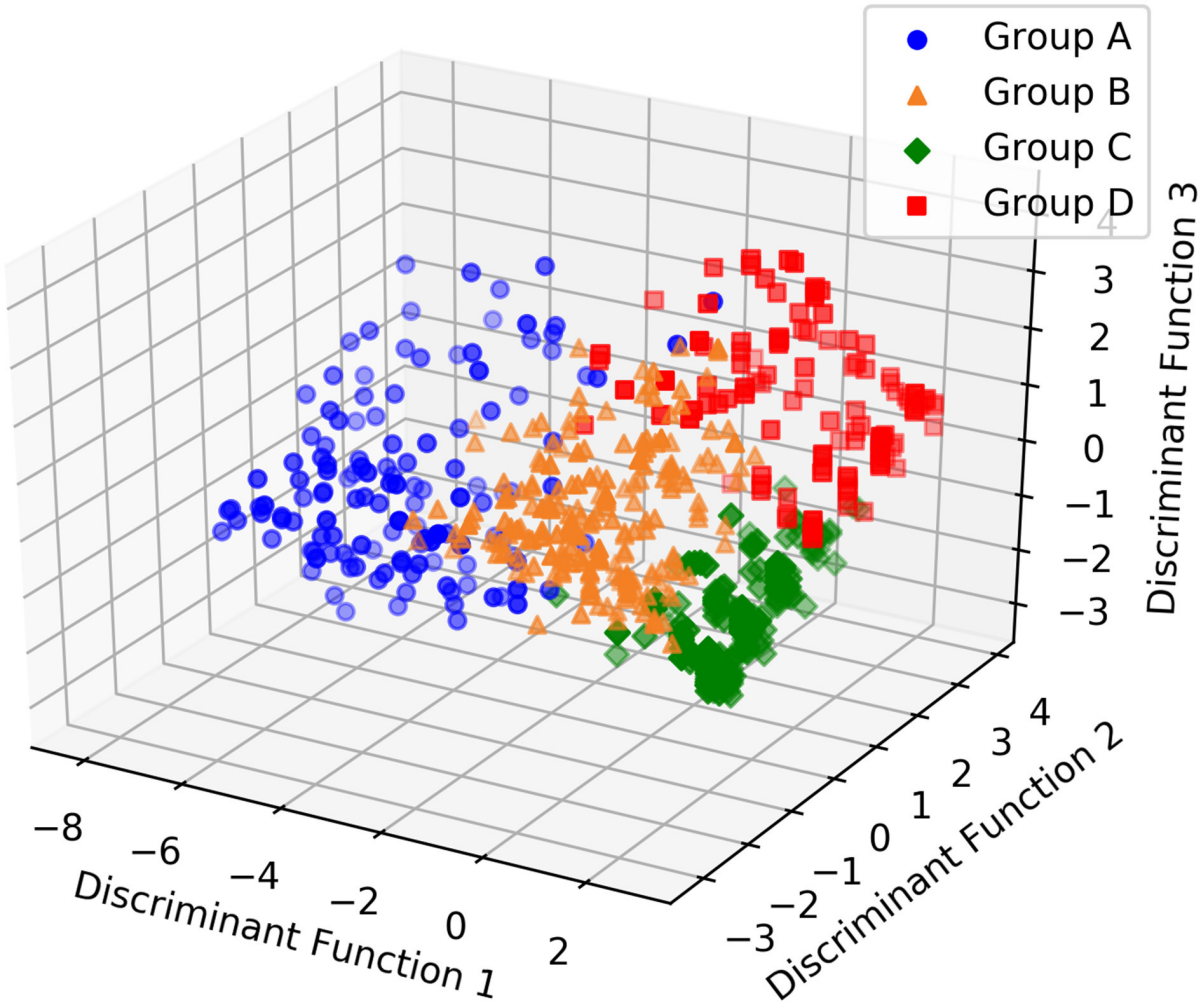
3D scatterplot showing how the four groups of dogs with separation related problems are separated by each other according to the three discriminant functions.

The discriminant analysis correctly assigned more than 90% of subjects overall (A 123/133, B 195/221, C 266/271, D 114/137; Confusion matrix: misclassification error = 8.4%; Table 6). Inspection of the discriminant function plots indicated that the first function seemed to clearly separate Cluster A (mean A = −5.32253) from the others, but especially Clusters C and D (mean B = 0.278996, C = 1.65767, D = 1.438027). The second function was most useful for separating Clusters B and D (mean A = 0.417468, B = −1.47841, C = −0.05577, D = 2.089912); while the third function seemed to be most important in separating B and D from C and to a lesser extent A (Mean A = −0.39897, B = 1.091871, C = −1.32817, D = 1.253233) (Figure 3 and Table 6).

**Table 6:** The three discriminant functions calculated to separate the four primary clusters of dogs with separation related problems (*n* = 762).

**Groups identified**	**Discriminant function number**	**Coefficient**	**Exit frustration**	**Social panic**	**Elimination**	**Redirected frustration**	**Reactive communication**	**Immediate frustration**	**Noise sensitivity**
A	1	2.069	−9.124	0.063	0.364	−0.961	−0.476	−0.291	2.997
B from D	2	3.608	2.298	−0.062	0.226	−2.578	−4.189	0.774	−2.517
B and D from C	3	0.956	−2.545	−0.759	−0.282	3.872	−2.429	−0.223	1.532

### Subpopulation Profiles Within Individual Population Clusters

Using the same process as before, Clusters A, B, and C were each composed of three sub-clusters and Cluster D of 2 sub-clusters. HACA and CAPAM provided similar solutions for the sub-clusters within A, C, and D, but there was less consistency for Cluster B (see Supplementary Material). The PC score profiles for the individual sub-clusters identified using HACA are described in Tables 7–10 below, with each followed by the results from the statistical analysis aimed at distinguishing between the relevant sub-clusters.

**Table 7:** Average principal component score followed by standard deviation (SD) of each sub-cluster identified with HACA between sub-clusters A.

**Principal components**	***N***	**Average scores and SD of subgroups of group A**		
		**A1 (*N*_**t**_ = 42)**	**A2 (*N*_**t**_ = 47)**	**A3 (*N*_**t**_ = 44)**
**Exit frustration**	*N*_t_	0.717 ± 0.205	0.670 ± 0.12	0.743 ± 0.137
	*N_*s*_*	*0.717 ± 0.205* *(N_*s*_ = 42)*	*0.670 ± 0.12* *(N_*s*_ = 47)*	*0.743 ± 0.137* *(N_*s*_ = 44)*
**Social panic**	*N*_t_	0.532 ± 0.28^a^	0.739 ± 0.147^b^	0.461 ± 0.244^a^
	*N_*s*_*	*0.532 ± 0.28* *(N_*s*_ = 42)*	*0.739 ± 0.147* *(N_*s*_ = 47)*	*0.461 ± 0.244* *(N_*s*_ = 44)*
**Elimination**	*N*_t_	0.107 ± 0.21	0.255 ± 0.309	0.221 ± 0.263
	*N_*s*_*	*0.346 ± 0.247* *(N_*s*_ = 13)*	*0.445 ± 0.286* *(N_*s*_ = 27)*	*0.441 ± 0.199* *(N_*s*_ = 22)*
**Redirected frustration**	*N*_t_	0.794 ± 0.164^a^	0.862 ± 0.168^a^	0.432 ± 0.195^b^
	*N_*s*_*	*0.794 ± 0.164* *(N_*s*_ = 42)*	*0.862 ± 0.168* *(N_*s*_ = 47)*	*0.432 ± 0.195* *(N_*s*_ = 44)*
**Reactive communication**	*N*_t_	0.394 ± 0.242^a^	0.896 ± 0.164^b^	0.851 ± 0.163^b^
	*N_*s*_*	*0.447 ± 0.205* *(N_*s*_ = 37)*	*0.896 ± 0.164* *(N_*s*_ = 47)*	*0.851 ± 0.163* *(N_*s*_ = 44)*
**Immediate frustration**	*N*_t_	0 ± 0	0 ± 0	0 ± 0
	*N_*s*_*	*0 ± 0* *(N_*s*_ = 0)*	*0 ± 0* *(N_*s*_ = 0)*	*0 ± 0* *(N_*s*_ = 0)*
**Noise sensitivity**	*N*_t_	0 ± 0	0.005 ± 0.037	0.006 ± 0.038
	*N_*s*_*	*0 ± 0* *(N_*s*_ = 0)*	*0.25 ± 0* *(N_*s*_ = 1)*	*0.098 ± 0.133* *(N_*s*_ = 3)*

*N_t_, all subjects in that cluster; N_s_, subjects showing at least one of the signs in that cluster for a given principal component. Bold PCs are significantly different within the cluster, with different superscript letters identifying the source of difference, all p <0.001. Italic values are the total number of dogs that presented at least one of the signs in that cluster for a given principal component*.

**Table 8:** Average principal component score followed by standard deviation (SD) of each sub-cluster identified with HACA between sub-clusters B.

**Principal components**	***N***	**Average scores of subgroups of group B**		
		**B1 (*N*_**t**_ = 103)**	**B2 (*N*_**t**_ = 78)**	**B3 (*N*_**t**_ = 40)**
**Exit frustration**	*N*_t_	0.109 ± 0.13	0.078 ± 0.111	0.095 ± 0.99
	*N_*s*_*	*0.181 ± 0.125* *(N_*s*_ = 62)*	*0.169 ± 0.106* *(N_*s*_ = 36)*	*0.165 ± 0.071* *(N_*s*_ = 23)*
**Social panic**	*N*_t_	0.579 ± 0.175^***^^a^	0.147 ± 0.12^***^^b^	0.477 ± 0.286^***^^a^
	*N_*s*_*	*0.579 ± 0.175* *(N_*s*_ = 103)*	*0.188 ± 0.103* *(N_*s*_ = 61)*	*0.502 ± 0.27* *(N_*s*_ = 38)*
**Elimination**	*N*_t_	0.106 ± 0.181^***^^a^	0.041 ± 0.092^***^^a^	0.61 ± 0.21^***^^b^
	*N_*s*_*	*0.321 ± 0.174* *(N_*s*_ = 34)*	*0.2 ± 0.097* *(N_*s*_ = 16)*	*0.61 ± 0.21* *(N_*s*_ = 40)*
**Redirected frustration**	*N*_t_	0.629 ± 0.237^***^^a^	0.724 ± 0.174^**^^b^	0.842 ± 0.141^***^^c^
	*N_*s*_*	*0.629 ± 0.237* *(N_*s*_ = 103)*	*0.724 ± 0.174* *(N_*s*_ = 78)*	*0.842 ± 0.141* *(N_*s*_ = 40)*
**Reactive communication**	*N*_t_	0.807 ± 0.192	0.835 ± 0.22	0.865 ± 0.159
	*N_*s*_*	*0.807 ± 0.192* *(N_*s*_ = 103)*	*0.835 ± 0.22* *(N_*s*_ = 78)*	*0.865 ± 0.159* *(N_*s*_ = 40)*
**Immediate frustration**	*N*_t_	0 ± 0	0 ± 0	0.025 ± 0.158
	*N_*s*_*	*0 ± 0* *(N_*s*_ = 0)*	*0 ± 0 (N_*s*_ = 0)*	*1.0 ± 0.0* *(N_*s*_ = 1)*
**Noise sensitivity**	*N*_t_	0.036 ± 0.166	0.006 ± 0.04	0.006 ± 0.04
	*N_*s*_*	*0.75 ± 0.177* *(N_*s*_ = 5)*	*0.25 ± 0.0* *(N_*s*_ = 2)*	*0.25 ± 0.0* *(N_*s*_ = 1)*

*N_t_, all subjects in that cluster; N_s_, subjects showing at least one of the signs in that cluster for a given principal component. Bold PCs are significantly different within the cluster, with different superscript letters identifying the source of difference*,

** p <0.01;

*** *p <0.001. Italic values are the total number of dogs that presented at least one of the signs in that cluster for a given principal component*.

**Table 9:** Average principal component score followed by standard deviation (SD) of each sub-cluster identified with HACA between sub-clusters C.

**Principal components**	***N***	**Average scores of subgroups of group C**		
		**C1 (*N*_**t**_ = 128)**	**C2 (*N*_**t**_ = 27)**	**C3 (*N*_**t**_ = 116)**
**Exit frustration**	*N*_t_	0.009 ± 0.036	0 ± 0	0.005 ± 0.035
	*N_*s*_*	*0.138 ± 0.052* *(N_*s*_ = 8)*	*0 ± 0* *(N_*s*_ = 0)*	*0.2 ± 0.1* *(N_*s*_ = 3)*
**Social panic**	*N*_t_	0.618 ± 0.18^**^^a^	0.447 ± 0.256^**^^b^	0.182 ± 0.141^***^^c^
	*N_*s*_*	*0.618 ± 0.18* *(N_*s*_ = 128)*	*0.483 ± 0.23* *(N_*s*_ = 25)*	*0.224 ± 0.122* *(N_*s*_ = 94)*
**Elimination**	*N*_t_	0.094 ± 0.146^***^^a^	0.734 ± 0.107^***^^b^	0.129 ± 0.173^***^^a^
	*N_*s*_*	*0.273 ± 0.115* *(N_*s*_ = 44)*	*0.734 ± 0.107* *(N_*s*_ = 27)*	*0.31 ± 0.126* *(N_*s*_ = 48)*
**Redirected frustration**	*N*_t_	0.041 ± 0.078	0.012 ± 0.045	0.052 ± 0.102
	*N_*s*_*	*0.178 ± 0.042* *(N_*s*_ = 30)*	*0.167 ± 0.0* *(N_*s*_ = 2)*	*0.222 ± 0.08* *(N_*s*_ = 27)*
**Reactive communication**	*N*_t_	0.852 ± 0.185	0.91 ± 0.132	0.844 ± 0.15
	*N_*s*_*	*0.852 ± 0.185* *(N_*s*_ = 128)*	*0.91 ± 0.132* *(N_*s*_ = 27)*	*0.844 ± 0.15* *(N_*s*_ = 116)*
**Immediate frustration**	*N*_t_	0.005 ± 0.059	0 ± 0	0 ± 0
	*N_*s*_*	*0.167 ± 0.0* *(N_*s*_ = 1)*	*0 ± 0* *(N_*s*_ = 0)*	*0 ± 0* *(N_*s*_ = 0)*
**Noise sensitivity**	*N*_t_	0.002 ± 0.022	0 ± 0	0.002 ± 0.023
	*N_*s*_*	*0.25 ± 0.0* *(N_*s*_ = 1)*	*0 ± 0* *(N_*s*_ = 0)*	*0.25 ± 0.0* *(N_*s*_ = 1)*

*N_t_, all subjects in that cluster; N_s_, subjects showing at least one of the signs in that cluster for a given principal component. Bold PCs are significantly different within the cluster, with different superscript letters identifying the source of difference*,

** p <0.01;

*** *p <0.001. Italic values are the total number of dogs that presented at least one of the signs in that cluster for a given principal component*.

**Table 10:** Average principal component score followed by standard deviation (SD) of each sub-cluster identified with HACA between sub-clusters D.

**Principal components**	***N***	**Average scores of subgroups of group D**	
		**D1 (*N*_**t**_ = 88)^**A**^**	**D2 (*N*_**t**_ = 49)^**B**^**
**Exit frustration**	*N*_t_	0.013 ± 0.048^***^	0.08 ± 0.1^***^
	*N_*s*_*	*0.157 ± 0.079* *(N_*s*_ = 7)*	*0.15 ± 0.091* *(N_*s*_ = 26)*
**Social panic**	*N*_t_	0.248 ± 0.218^**^	0.381 ± 0.239^**^
	*N_*s*_*	*0.341 ± 0.184* *(N_*s*_ = 64)*	*0.424 ± 0.212* *(N_*s*_ = 44)*
**Elimination**	*N*_t_	0.116 ± 0.228	0.19 ± 0.251
	*N_*s*_*	*0.464 ± 0.217* *(N_*s*_ = 22)*	*0.443 ±* 0.183 *(N_*s*_ = 21)*
**Redirected frustration**	*N*_t_	0.072 ± 0.11^***^	0.694 ± 0.181^***^
	*N_*s*_*	*0.211 ± 0.075* *(N_*s*_ = 30)*	*0.694 ± 0.181* *(N_*s*_ = 49)*
**Reactive communication**	*N*_t_	0.220 ± 0.181	0.170 ± 0.178
	*N_*s*_*	*0.303 ± 0.14* *(N_*s*_ = 64)*	*0.298 ± 0.129* *(N_*s*_ = 28)*
**Immediate frustration**	*N*_t_	0 ± 0	0 ± 0
	*N_*s*_*	*0 ± 0* *(N_*s*_ = 0)*	*0 ± 0* *(N_*s*_ = 0)*
**Noise sensitivity**	*N*_t_	0.003 ± 0.027	0.005 ± 0.036
	*N_*s*_*	*0.093 ± 0.136* *(N_*s*_ = 3)*	*0.25 ± 0.0* *(N_*s*_ = 1)*

*N_t_, all subjects in that cluster; N_s_, subjects showing at least one of the signs in that cluster for a given principal component. Bold PCs are significantly different within the cluster, with different superscript letters identifying the source of difference*,

** p <0.01;

*** *p <0.001. Italic values are the total number of dogs that presented at least one of the signs in that cluster for a given principal component*.

Using the data from the HACA, the three sub-clusters within A (A1 *n* = 42, A2 *n* = 47, and A3 *n* = 44), had A2 scoring significantly higher for social panic, A3 lower for redirected frustration and A1 significantly lower for reactive communication than the other two sub-clusters (all *p* < 0.001). The flexible discriminant analysis correctly assigned 87.93% of subjects to these sub-clusters. Inspection of the discriminant function plots indicated that the first function seemed to clearly separate sub-cluster A1 from A3 (means A1 = −1.939, A2 = 0.206, A3 = 1.630); the second function was most useful for separating A2 from A3 and to a lesser extent A1 (mean A1 = 0.726, A2 = −1.626, A3 = 1.044; Table 11).

**Table 11:** The discriminant functions calculated to distinguish between the sub-clusters of dogs within each primary cluster.

**Sub-groups identified**	**Discriminant function number**	**Coefficient**	**Exit frustration**	**Social panic**	**Elimination**	**Redirected frustration**	**Reactive communication**	**Immediate frustration**	**Noise sensitivity**
A1 from A3	1	−1.689	1.174	0.498	1.219	−4.001	4.286	5.168	0.0
A2 from A3 and A1	2	5.086	1.810	−2.573	−0.328	−3.951	−2.836	−1.763	0.0
B2 from B3	1	−1.529	−0.909	3.153	5.18	0.428	−0.341	−0.909	0.592
B1 from B2 and B3	2	1.627	−0.192	5.066	−3.221	−3.462	−0.882	0.714	1.899
C1 from C2	1	−0.583	−0.414	−3.054	5.815	−0.946	1.034	1.255	1.618
C from C3	2	3.714	−0.072	−4.98	−3.021	1.294	−1.388	1.175	−1.503
D1 from D2	1	−2.148	−1.676	1.548	−1.136	7.981	−2.078	−4.189	0.0

The three sub-clusters within B (B1 *n* = 103, B2 *n* = 78, B3 *n* = 40) differed in their PCA profile, with B3 scoring significantly higher for social panic and elimination than the other two sub-clusters and the three clusters scoring significantly differently for redirected frustration, which was highest within B3 and lowest in B1. The discriminant function analysis correctly allocated 90.5% of subjects, with the first function most clearly separating B2 and B3 (mean B1 = 0.225, B2 = −1.514, B3 = 2.374) and the second one B1 from B2 and B3 (mean B1 = 1.379, B2 = −1.007, B3 = −1.588; Table 11).

The three sub-clusters within C (C1 *n* = 128, C2 = 27, C3 = 116) differed in their PCA profile, with each cluster significantly different in its social panic score (C1 highest, C3 lowest *p* < 0.01) and C2 significantly lower in its elimination score. The discriminant function analysis correctly allocated 91.9% of subjects, with the first function most clearly separating C1 from C2 (Mean C1 = −1.078, C2 = 3.246, C3 = 0.434) and the second function most clear separating C2 from C3 (mean C1 = −0.774, C2 = −1.974, C3 = 1.314; Table 11).

The two sub-clusters of D (D1 *n* = 88, D2 *n* = 49) differed in the PC profile with D1 significantly lower than D2 in its exit frustration, social panic and redirected frustration scores (all *p* < 0.001). The discriminant function analysis correctly allocated 98.5% of subjects, with the function (mean D1 = −1.813, D2 = 3.255; Table 11).

## Discussion

This study used data from the largest recorded sample of dogs presenting with some form of separation related problem. The broad definition used for inclusion was based on the presence of one or more cardinal signs which is in line with what is widely used elsewhere [e.g., (4, 7, 8, 12, 18–21, 36, 37, 65)] but included a larger number of routine behaviors not yet studied. The finding that the sample used in the analysis (762) was generally representative of the wider population for which we had completed surveys (2,757) indicates bias was largely reduced; this together with the quality checks built into our analysis (e.g., split sample PCA confirmation) gives us confidence in the robustness and validity of these results. With the PCA, we have defined for the first time the main behaviors contributing to the variation in signs associated with separation related problems; with the cluster analysis we have identified for the first time, how there appear to be four main forms of the condition, for which we can infer specific and distinct psychological causes. Our subsequent analyses provide further ideographically important information for clinicians that will enable them to make more reliable diagnoses, and so propose more specific treatments. The current work is focused on describing norms for different forms of separation related problems and the creation of a more robust diagnostic pathway, and so we discuss these further below. Treatment considerations are outside of the scope of the current study, but our framework will allow the development and empirical testing of treatment hypotheses based on solid ground truths.

### The Methodology Used

Using a bottom up approach without any predefined framework [e.g., (15)] the dataset was not constrained by any theory that might bias interpretation of the results. We used a broad definition of separation related problems based on the presence of one or more of the cardinal signs described in the literature, and recruited from the general dog population, rather than individuals seeking assistance with the problem or some other criterion, as has been used in other studies. As we did not wish to bias the results on the basis of predefined theoretical perspectives, we asked about a wide range of signs within the survey covering a spectrum of potential underlying motivations and emotions that might result in such separation related problems [q.v. (29)]. Thus, it allowed a comprehensive assessment of the mathematical relationship between all signs of potential importance in order to disambiguate SRP signs in adolescent and adult dogs [c.f. (21)]. By doing so, the PCA not only confirmed the importance of both some well-recognized and common signs such as destructiveness and vocalization (13, 30), but also highlighted how some well-recognized signs might be relatively uncommon, e.g., fear responses to noises, and do not contribute to a unique form of SRPs despite previous opinion to the contrary (15, 66). Likewise, the importance of distinguishing between destruction of objects associated with the owner or their scent, emphasized by some from a theoretical perspective (15) was not supported by this first empirical test of that hypothesis. Instead, destructiveness tended to be differentiated largely on the basis of whether it was carried out with the claws and directed toward the exit (PC1) or apparently with the mouth and directed toward medium sized objects in the home (but not those especially associated with the owner, such as their favorite seat).

The wide range of signs here considered also allowed us to demonstrate the importance of previously unknown albeit rare signs in the variability of forms of separation related problems (SRPs), such as aggression toward the owner at certain times when they try to restrain the dog.

As a further precaution against bias, we avoided imputing values where data were missing and gave owners the option to indicate that they did not know. This resulted in a high rate of attrition between recruitment and initial data analysis, however, the final sample of 762 dogs was comparable to the wider sample of 2,757 completed questionnaires, with a good gender split, and no particular breed dominating the dataset. It was surprising to see Weimaraners among the most popular breeds in our survey as it is not listed in the top 10 “most popular” breeds in either the UK or USA. It is uncertain whether the high prevalence reflects an increased risk in this breed [although this has not been identified in other studies focused on clinical caseloads e.g., (11, 12, 25)] or is a product of the recruitment method used, since we are aware that some breed societies promoted the survey to their members. By assessing the data in two stages, initially with 345 subjects who had complete datasets without any “don't know” responses, and then returning to the sample to identify a further 417 subjects who had complete data for the 56 items identified as most important for explaining the variation in the initial data, we were able to in effect undertake a split sample test for reliability. The structure in the two samples was similar indicating that there was no significant bias in the samples, and the results were robust. We were then able to pool the data, to create the final dataset used for all subsequent analyses. The use of the two forms of cluster analysis assessed the reliability of the results at this level of the process, and the two processes converged extensively. We know little about the reliability of veterinary behavior diagnoses, and this has been a longstanding issue in human psychiatry (67). However, the overall reliability of 90% in the assignment of individuals to clusters compares favorably with the 0.84 Kappa coefficient value considered excellent for test-retest diagnosis of separation anxiety disorder in humans using the Anxiety Disorders Interview Schedule for DSM-IV (68). These findings, which are based on the largest sample of dogs with separation related problems to date, give us confidence in the robustness of the latent structure to the problem that we have identified.

### The Main Behaviors Consistently Describing and Distinguishing Different Forms of Separation Related Problem

The rank order frequency of signs (vocalization> destructiveness> elimination) is similar to that reported by some authors [e.g., (4, 12, 28, 30)], but differs from that reported by others [e.g., (25, 39, 69)]. However, few have considered the prevalence of depression-like signs, which in our sample had the second highest prevalence of the cardinal signs at 52.9%. The suggestion that SRPs may often reflect a depressive-like state, is supported by clinical work using cognitive judgement tasks in affected subjects (18). This sign may easily be overlooked by owners, and while not appearing important in differentiating forms of SRP, should be noted for its animal welfare significance and in order to help owners better understand the condition affecting their dog. It is possible that one of the reasons why depression-like signs did not help to disambiguate SRPs is because it is seen as a general state and not defined well by specific context (15 contexts featured depression like responses in the original survey); by contrast, signs like vocalization and destructiveness were included in many items on the basis of their occurrence in different contexts and these featured in different principal components. Furthermore, depression-like signs are subjective and might need better definition in order to be of diagnostic value. In addition, it might be argued that “Looking depressed/sad” is highly subjective, and might depend on the owners' emotionality, empathy, and sense of guilt at leaving their dogs alone, which could easily over-estimate SRP. The widespread prevalence of this sign (it was the second most common one in our sample) together with its poor specification might explain why it was not useful in explaining the variance in our sample. It was somewhat surprising that items relating to elimination occurred together in a single principal component, given the diverse contexts in which it could occur, including in the presence of the owner. Signs of depression and salivation did not load on any PC, despite their inclusion in the diagnosis of “separation anxiety” (37, 43, 65, 69, 70). In total 54 signs, grouped into seven principal components defined the variation in the population, which was structured into four clusters, and 11 sub-clusters. We consider these the four primary forms of SRP in dogs, and eleven more tightly defined phenotypes and discuss them further in the next section.

The principal components derived from the PCA indicate that the expression of frustration by dogs in different contexts appears to be very important to the variability of SRPs. Three of the components (PC1- exit frustration, PC4- redirected frustration and PC6- immediate frustration) seem to relate to frustration, explaining 24% of the total variance in the presenting complaint, with a potential fourth component as well (PC5—reactive communication), explaining a further 6%. The latter included barking vs. more friendly greetings toward visitors and more widespread reactivity to those intruding into the dog's personal space [recognized as a trigger of frustration or Rage sensu Panksepp (64)], and so might reflect a form of frustration intolerance by the dog (71). This interpretation is perhaps more equivocal and it might be argued that it instead reflects a more general sociability or reactivity. The recent development of a frustration assessment instrument in dogs (71), may allow further elucidation on this point, but we note that the development of protocols specifically described as being focused on frustration tolerance are rare in the veterinary behavior literature (72), and have not to our knowledge been specifically described in relation to the contexts described here. This is clearly an important gap in the treatment literature. The emphasis on the potential role of frustration in SRP is in contrast to previous work which has tended to focus on the significance of the bond between the dog and owner (7, 15, 36, 38, 73, 74) and/or fears, especially as a result of association with unpleasant noisy events (15, 66, 75). Both of these featured here (PC2—social panic; PC7—noise sensitivity) and it should be noted the two may not be exclusive from an ontogenetic perspective. For example, in humans (41, 76), it has been noted that certain forms of maternal caregiving styles (i.e., Ambivalent, in which the carer is emotionally inconsistent toward their child and Disorganized in which the carer is extremely erratic) result in equivalent insecure forms of attachment in the child which are characterized by varying degrees of frustration. Ambivalently attached children are typically insecure anxious and angry, while Disorganized attached children are often depressed and angry. Thus, it might be that owner caring style plays an important role in generating emotional predispositions that increase the risk of the dog having difficulties when faced with the frustration of being contained when the owner leaves. The importance of this is the subject of a future publication being prepared by the authors and in line with the growing evidence which indicates that these dogs are not overattached to their owners (19, 26, 28, 74). Indeed, potential signs of hyperattachment, previously thought to be important in differentiating SRPs, like following the owner around and attention seeking or destroying areas strongly associated with the owner or their scent (15, 73), were not important in differentiating different forms of SRP. It is worth noting that although most of the PCs seem to relate to specific forms of emotional arousal consistent with the affective states described by Panksepp [(64), we use the term frustration in preference to RAGE, to avoid confusion], some seem to relate to arousal in quite specific contexts such as loud noises or the arrival of visitors/presence of strangers. Nonetheless, as already discussed, their casual significance as triggers of SRP cannot be determined in a study such as this. Indeed, it is worth noting that some of the putative states identified here, e.g., exit frustration, may have diverse triggers, and a priori assumptions about underlying motivation may be misleading. Thus, exit frustration might arise from affective states related to a desire to escape from something perceived to be aversive in the home [FEAR sensu Panksepp, (64)], a need to be with the owner as a result of their attachment-related dependence (PANIC) or attraction to/interest in other stimuli outside (SEEKING/ desire) (29), or reinforcement of such behavior through a reduction in the frequency or duration of being left alone. It is also worth noting at this point that not only was an association between noise sensitivity and SRP rare, but also, this PC was not particularly associated with any specific cluster, indicating that fear of noises does not seem to result in a particular form of SRP from a motivational-emotional perspective. We discuss further the potential causal pathways for the different forms of SRP below, as we consider the nature of the clusters identified.

### The Grouping of Signs to Describe Specific Forms of Separation Related Problems and Their Interpretation

Given that the groups identified using the hierarchical agglomerative method (HACA) showed good agreement with those defined using the partitioned around medoid method (CAPAM), we use the groups as defined by HACA as the basis for our discussion, focusing especially on the interpretation of Tables 5, 7–10. We propose a range of explanations for the clusters of signs by way of initial hypotheses from which deductions might be made and tested in future work, in order to gain greater insight into the nature of these different forms of SRP.

#### Cluster A

Cluster A is the smallest group (17.4% of the population) characterized by all members showing signs of exit frustration at a relatively high level (the average value in Table 5 equates to a little more than seven of the 10 signs included in this PC), social panic and redirected frustration (at relatively high levels—around 9/15 and 4/6 signs, respectively), but no signs of immediate frustration toward the owner. We propose that the most parsimonious explanation for this PC profile is that these dogs find being separated aversive (social panic signs) and try to go after the owner (exit frustration), but, because they are unable to do so due to barriers within the home, they struggle to find an alternative way of coping (redirected frustration). They do not appear hostile to their owners (immediate frustration). We propose this cluster be referred to as “*exit frustration” related SRPs* until such time as empirical evidence elucidates a clearer alternative cause.

Cluster A is relatively evenly divided between three sub-clusters, which differ on the basis of their average social panic scores (highest in A2, with around 11/15 signs shown, and lowest in A3 with only about 7/15 signs shown on average, and 8/15 signs in A1), their average redirected frustration scores (averaging around 5/6 signs for A1 and 5-6/6 for A2, but only 3/6 signs for A3) and their reactive communication scores (averaging only 0 from the range −2 to +4 of signs for A1, but +3 or +4 for A2 and A3). The latter was not a defining feature of cluster A but is clearly of importance in differentiating its subtypes since A1 is markedly less reactive and this appears to set it apart from A2 and A3. Indeed, A2 appears to contain the most severely affected dogs with high levels of social panic, both types of destruction and a high level of reactivity. Whereas, A1 tends to destroy both exits and medium sized objects, A3 appears to be more focused on the destruction of the exit. It is also worth noting that within cluster A there appears to be a relationship between social panic and redirected frustration scores; while this could be coincidental, it should be investigated further whether an inability to cope in this situation (as expressed by redirected frustration) is associated with suboptimal forms of attachment (as evidenced by increasing signs of social panic). According to Panksepp et al. (77), interactions between a mother and child that result in a problematic attachment style (e.g., Disorganized attachment) do not allow the child to easily regulate his/her emotions, and therefore the child tends to be more sensitized to subsequent distress such as the frustration of not accessing a desired resource (including their mothers) enhancing the chances of anger being triggered in these contexts.

#### Cluster B

Cluster B makes up 29% of the population and is characterized by all members showing redirected frustration and reactive communication at relatively high levels (4-5/6 signs and +3 from the range −2 to +4 of signs, respectively), nearly all showing signs of social panic (6/15 signs), with about three quarters showing signs of exit frustration but at a low level (on average just about one sign for those that show any of the signs in this PC). Although immediate frustration toward the owner is a very rare phenomenon in SRP, this is one of the groups which does feature it. We propose that the most parsimonious explanation for this collection of PCs is that these dogs are agonistically reactive to external events (reactive communication), and often try to get at these stimuli as an immediate response (exit frustration) but because they are unable to do so due to barriers within the home, they remain highly aroused and struggle to find an alternative way of coping (redirected frustration). Accordingly, these dogs find being separated from the owner aversive (social panic signs), with the rare instances of aggression toward the owner (immediate frustration) being indicative of a more general agonistic reactivity. We propose this cluster be referred to as “*redirected reactive*” *related SRPs* until such time as empirical evidence elucidates a clearer alternative cause.

The three sub-clusters making up cluster B are of quite different sizes (103, 78, and 40 dogs, respectively) and differ from each other on the basis of their redirected frustration scores (B1 < B2 < B3). B2 is further characterized by significantly lower social panic scores; while B3 is characterized by higher levels of elimination and all subjects showing at least one sign (compared to about a third and fifth in B1 and B2, respectively). The significance of elimination in differentiating within this cluster is interesting since it is a sign that has long been speculated to be indicative of emotionality (78) and the initial interpretation of Cluster B was based on these animals being quite reactive to external events. Elimination has been associated with high arousal states (79, 80) and stress mediated by sympathetic activity in the locus coeruleus (81) and this would suggest that dogs within B3 may be particularly reactive on the basis of the frequency of exposure to eliciting events (82). Although rare overall as a presenting feature, it is worth noting that B3 was the sub-cluster most frequently associated with signs of immediate frustration toward the owner (7.5% of cases). We speculate, given this convergence of signs, that interventions specifically focused on controlling the emotionality/reactivity of dogs in this cluster may be of particular value in helping to reduce the risk of redirected frustration when associated with elimination; reducing impulsivity may be particularly valuable for those in sub-cluster B3. These hypotheses deserve empirical investigation through targeted clinical trials using the classification described here. Dogs within B1 show relatively high levels of social panic but relatively few signs of elimination, which may be more consistent with dominance of the hypothalamo-pituitary adrenal axis due to more chronic exposure stress. Indeed, the relatively high level of signs of social panic, inconsistent signs of redirected frustration, and high levels of reactive communication of B1 appear superficially but remarkably similar to the signs of Disorganized attachment described in children (83). We speculate that this may be one form of SRP that is closely related to the form of bond between dog and owner. Again, this deserves further empirical investigation. B1 also shows the higher number of dogs with higher scores on noise sensitivity (five dogs with average 0.75, SD 0.177) than any other subgroup, which might help explain the very high reactivity of these dogs. According to Jones and Gosling (84), both “reactivity” and “fearfulness” aspects of temperament are often related, and this may be pertinent to the evaluation of the development of the problem in these cases.

#### Cluster C

Cluster C is the largest group (35.6% of the population) and is characterized by all members showing signs of reactive communication at relatively high levels (about +3 from the range −2 to +4 of signs) and nearly all showing signs of social panic (at relatively moderate levels−5-6/15 signs on average). Signs of exit frustration are rare (<5% of dogs) and redirected frustration uncommon (21.7%) and at a low level when it does occur. Although immediate frustration toward the owner is a very rare phenomenon in SRP, this is the other group which features it. We propose that the most parsimonious explanation for this collection of PCs is that these dogs are reactive to external events (reactive communication), but unlike cluster B, dogs do not typically try to get at these stimuli (exit frustration). This might be, for example, because they are more generally anxious and avoidant than dogs in Cluster B. This would be consistent with the absence of the owner being associated with a loss of social support (social panic) but the dogs perhaps finding some safety in the home, reducing their arousal (redirected frustration). The rare instances of aggression toward the owner (immediate frustration) might be indicative of a more extreme nervousness. We propose this cluster be referred to as “*reactive inhibited*” *related SRPs* until such time as empirical evidence elucidates a clearer alternative cause.

C2 is markedly smaller (*n* = 27) than the other two sub-clusters (128 and 116), and characterized by a higher level of elimination. The potential significance of the relationship between this PC, the potential involvement of sympathetic arousal and the frequency of exposure to reacting signs has been discussed in relation to cluster B, but it is worth noting that elimination signs again appear to be important for differentiating the other cluster of SRP that seems to relate to high levels of reactivity. Once again, one sub-cluster (C2) stands out on the basis of both the number of signs and the finding that all dogs within this sub-cluster show at least one sign, while only about a third of dogs in the other two sub-clusters show any signs of elimination at all. The three sub-clusters differ from each other in social panic, with signs being relatively uncommon in C3; not only are they highest in C1, but all dogs in this cluster showed at least 1 sign, unlike the other two—although nearly all showed at least one sign. Although C1 shows significantly greater anxiety at the anticipated departure of the owner compared to C2, it should be noted that there are a relatively large number of signs in C2, unlike C3, which is notable for the infrequency of these signs. It might be that the lack of signs of social panic reflect an earlier development stage in which the dog has not learned to anticipate the events following the owner's departure. Unlike cluster A, there is clearly no relationship between redirected frustration and social panic, and the significance of this needs to be noted when considering potential causal relationships between the two, which may be quite different between clusters A and C. In this regard, it is worth noting that Solomon and colleagues (85) have recently found that dogs with a higher score for an “Active/excitable” personality type (which might be analogous to the profile described in cluster C) are at a significantly greater risk of having an insecure attachment toward their caregiver. Thus, it might be that the type of attachment shown by dogs toward their owners is particularly important to the risk of this type of SRP. Sub-group C3 seem to be very reactive dogs that might respond to external stimuli when alone or not because they do not seem to try to avoid/become stressed with the separation from their owners (i.e., owners do not serve as a social support). C2 shows high elimination as a possible response to the high arousal. On the other hand, C1 dogs seem to be the opposite of C3 in being reactive both alone and with the owner but seeing their owner as a social support. Accordingly, both sub-clusters C1 and C2 are more likely to present insecure attached dogs, and consequently insecure caregivers (85).

#### Cluster D

Cluster D makes up 18% of the population and is characterized by a lack of consistency in signs across all members, although none show signs of immediate frustration toward the owner; exit frustration is also relatively rare (24% of dogs). Social panic is the most frequently shown group of signs (78.8%) although the number of signs is significantly less than the other clusters, with redirected frustration and reactive communication shown by a majority of subjects, albeit the latter with a relatively low number of signs. We propose that the most parsimonious explanation for this collection of PCs is that these dogs have learned that being alone is aversive (social panic) due to a lack of stimulation. This leads them to become more reactive to external events as time progresses [as described by Lund and Jørgensen (13)], which may ultimately relate in redirected frustration, as they cannot escape. We suggest that this cluster be referred to as “*boredom” related SRPs* [a term that has already been used to describe a form of SRPs by other authors, e.g., (14)], until such time as empirical evidence elucidates a clearer alternative cause.

The two sub-clusters of D differ significantly from each other in relation to three PC scores. Most notably, all subjects in D2 show at least one sign of redirected frustration (and typically around four or five signs on average from the total of six) but only about a third in D1 show any signs, and then only one or two. Exit frustration scores show a similar bias between the two sub-clusters, with signs from this PC being rare in D1, but occurring in about half of subjects in D2. However, in both sub-clusters, subjects who show any of these behaviors typically only show about 1/10 sign. The sub-clusters also differ in social panic score, although scores seem moderate (5-6/15 signs) and are quite variable between subjects, with nearly all subjects in D2 showing at least one sign of social panic, and around two thirds in D1. It seems that D1 contains the more relaxed dogs showing sporadic unwanted behaviors, while D2 seems to have dogs that become bored more frequently and have what some authors call a “lack of habituation to social isolation” [e.g., (39)], and therefore a higher aversive association with being alone.

### Allocating Individuals to Specific Clusters

Although we have described significant differences between clusters; it should be noted that this information is very much in line with a nomothetic approach to analysis. From a clinical perspective, an idiographic analysis is of more importance since it highlights what is necessary for differentiating different forms of SRP and allocating individuals appropriately to a given cluster or sub-cluster. Only if certain PCs are unique to, or perhaps uniquely absent from, certain clusters might it be reasonable to propose that they are of diagnostic value. If all subjects within a cluster show at least one of the signs of a given PC, then, should a patient present without any signs, it may be reasonable to consider it unlikely that the subject belongs within that cluster or sub-cluster. Likewise, if a sign never features in a given cluster or sub-cluster and a patient shows a given sign, then a similar deduction might be tempting. However, it should be considered that certain PCs occur quite rarely, and so their absence from the current dataset should not be used to make this inference with as much confidence as the former situation. This allows us to consider the necessary and/or sufficient conditions for the assignment of subjects to a given cluster (86). In this regard, signs of exit frustration, social panic and redirected frustration are necessary for a subject to belong to cluster A; with signs of reactive communicationalso necessary for it to be included in either A2 or A3. Signs of redirected frustration and reactive communication are necessary to include a subject in cluster B, with the signs of social panic required in addition for it to be included in B1. To be included in cluster C, subjects must show signs of reactive communication, and also signs of social panic to be included in C1 and signs of redirected frustration to be included in C2. There are no necessary prerequisites for D, but it could be argued that if a subject does not meet the necessary criteria described above for clusters A, B, and C, then by default it must belong to cluster D. Therefore, this could be considered a sufficient condition for assigning a subject to cluster D while there are no signs or conditions that are sufficient to assign it to either cluster A, B, or C. Beyond this, it is for the clinician to use the information described in the preceding sections to make a clinical judgement as to which cluster or sub-cluster a subject is most likely to belong to, given their presenting signs. While this remains partly clinical judgement, there is now, at least, an evidence base from which to draw. We will describe this process precisely in a future publication that will focus on the application of this framework in a clinical setting. The specific behavioral profile of the individual and its putative psychological basis will determine the precise treatment programme offered to an individual. It is unwise to suggest treatment programmes on the basis of broad cluster membership, since these will, by definition, be poorly contextualized and this is typical of the problems that arise when nomothetic information rather than idiographic knowledge is used as the basis of the formulation of treatment strategies for the individual a growing concern in human medicine (87). Nonetheless, this framework provides the first comprehensive basic ground truth data for separation related problems in dogs which should enable the formulation of more evidence-based hypotheses and guide researchers on how to select subjects more precisely in order to compare risk factors and formulate more effective treatments.

The definition of robust clusters in this way for the first time within veterinary behavioral medicine, provides a solid nosological basis for a deeper understanding of the nature of not only SRP but all behavior problems, that is grounded in data, rather than expert opinion. Future work will also be able to explore relevant aetiological, and differential treatment outcomes, in order to hopefully allow us to develop not only more effective treatment but also more effective prevention programmes.

## Data Availability Statement

The datasets generated for this study are available on request to the corresponding author.

## Ethics Statement

The animal study was reviewed and approved by College of Science Research Ethics Committee, University of Lincoln. Written informed consent was obtained from the owners for the participation of their animals in this study.

## Author Contributions

RM and DM designed the questionnaire and collected the answers. LA, DM, TP, and OB analyzed the data. LA and DM wrote the main manuscript text. All authors reviewed the manuscript.

### Conflict of Interest

The authors declare that the research was conducted in the absence of any commercial or financial relationships that could be construed as a potential conflict of interest.

## References

[1] Mearin F Ciriza C Mínguez M Rey E Mascort JJ Peña E. Clinical practice guideline : irritable bowel syndrome with constipation and functional constipation in the adult. Rev Esp Enfermidades Dig. (2016) 108:332–61. 10.17235/reed.2016.4389/2016 27230827

[2] Mair TS Howarth S Lane JG Evaluation of some prophylactic therapies for the idiopathic headshaker syndrome. Equine Vet J. (1992) 24:10–2. 10.1111/j.2042-3306.1992.tb04763.x 9109952

[3] Mills DS Conceptualising behaviour problems - separating a dog's bite from it's owner's problem. In: Mills D Heath SE Harrington LJ, editors. Proceedings of the first International Conference on Veterinary Behavioural Medicine. Birmingham: Universities Federation for Animal Welfare (1997).

[4] Bradshaw JWS Mcpherson JA Casey RA Larter S Larter IS. Aetiology of separation-related behaviour in domestic dogs. BMJ J. (2002) 151:43–6. 10.1136/vr.151.2.43 12148601

[5] Mills DS Mills CB A survey of the behaviour of UK household dogs. In: Proceedings of the 4th International Veterinary Behaviour Meeting. (2003). p. 93–8.

[6] Marques Soares G Telhado Pereira J Leal Paixâo R Estudo exploratorio da síndrome de ansíedade de separaçâo em caes de apartamento Exploratory study of separation anxiety syndrome in apartment dogs. Cienc Rural. (2010) 40:548–53. 10.1590/S0103-84782010000300008

[7] Borchelt PL Voith VL. Diagnosis and treatment of separation-related behavior problems in dogs. Vet Clin North Am Small Anim Pract. (1982) 12:625. 10.1016/S0195-5616(82)50106-4 6984556

[8] Wright JC Nesselrote MS Classification of behavior problems in dogs: distributions of age, breed, sex and reproductive status. Appl Anim Behav Sci. (1987) 19:169–78. 10.1016/0168-1591(87)90213-9

[9] Simpson BS Canine separation anxiety. Compend Contin Educ Pract Vet. (2000) 22:328–38.

[10] Denenberg S Landsberg GM Horwitz D Seksel K A comparison of cases referred to behaviorists in three different countries. In: Mills D Levine E Landsberg G Horwitz D, editors. Current Issues and Research in Veterinary Behavioral Medicine. Indiana: Purdue University (2005). p. 56–62.

[11] Bamberger M Houpt KA. Signalment factors comorbidity and trends in behavior diagnoses in dogs. J Am Vet Med Assoc. (2006) 229:1591–601. 10.2460/javma.229.10.1591 17107314

[12] Storengen LM Boge SCK Strøm SJ Løberg G Lingaas F A descriptive study of 215 dogs diagnosed with separation anxiety. Appl Anim Behav Sci. (2014) 159:82–9. 10.1016/j.applanim.2014.07.006

[13] Lund JD Jørgensen MC Behaviour patterns and time course of activity in dogs with separation problems. Appl Anim Behav Sci. (1999) 63:219–36. 10.1016/S0168-1591(99)00011-8

[14] Heath S Dealing with separation problems in dogs. In: Scientific Proceedings of the 45th Annual Congress of the British Small Animal Association. Gloucester: British Small Animal Veterinary Association (2002). p. 536–8.

[15] Appleby D Pluijmakers J. Separation anxiety in dogs: the function of homeostasis in its development and treatment. Clin Tech Small Anim Pract. (2004) 19:205–15. 10.1053/j.ctsap.2004.10.002 18371317

[16] Horwitz D Separation-related problems in dogs and cats. In: Horwitz D Mills D, editors. BSAVA Manual of Canine and Feline Behavioural Medicine. 2nd ed. Gloucester: British Small Animal Veterinary Association (2009).

[17] Basse C Blackwell EJ Bradshaw JWS Casey RA Predicting separation problems in dogs: development of a practical test for rehoming centers. J Vet Behav. (2010) 5:33–4. 10.1016/j.jveb.2009.10.021

[18] Karagiannis CI Burman OHP Mills DS Dogs with separation-related problems show a “less pessimistic” cognitive bias during treatment with fluoxetine (Reconcile^TM^) and a behaviour modification plan. BMC Vet Res. (2015) 11:1–10. 10.1186/s12917-015-0373-1 25889323 PMC4393593

[19] Konok V Kosztolányi A Rainer W Mutschler B Halsband U Miklósi Á. Influence of owners' attachment style and personality on their dogs' (*Canis familiaris*) separation-related disorder. PLoS ONE. (2015) 10:e0118375. 10.1371/journal.pone.0118375 25706147 PMC4338184

[20] van Rooy D Arnott ER Thomson PC McGreevy PD Wade CM Using an owner-based questionnaire to phenotype dogs with separation-related distress: do owners know what their dogs do when they are absent? J Vet Behav Clin Appl Res. (2018) 23:58–65. 10.1016/j.jveb.2017.10.009

[21] Konok V Marx A Faragó T Attachment styles in dogs and their relationship with separation-related disorder – a questionnaire based clustering _ Elsevier Enhanced Reader. Appl Anim Behav Sci. (2019) 213:81–90. 10.1016/j.applanim.2019.02.014

[22] Ogata N Separation anxiety in dogs: what progress has been made in our understanding of the most common behavioral problems in dogs? J Vet Behav Clin Appl Res. (2016) 16:28–35. 10.1016/j.jveb.2016.02.005

[23] Eysenck HJ Psychiatric diagnosis as a psychological and statistical problem. Psychol Rep. (1955) 1:3–17. 10.2466/pr0.1955.1.g.3

[24] Venkatasubramanian G Keshavan MS. Biomarkers in psychiatry – A critique. Ann Neurosci. (2016) 23:3–5. 10.1159/000443549 27536015 PMC4934408

[25] Flannigan G Dodman NH Risk factors and behaviors associated with SA in dogs. J Am Vet Med Assoc. (2001) 219:460–6. 10.2460/javma.2001.219.460 11518171

[26] McGreevy PD Masters AM Risk factors for separation-related distress and feed-related aggression in dogs: additional findings from a survey of Australian dog owners. Appl Anim Behav Sci. (2008) 109:320–8. 10.1016/j.applanim.2007.04.001

[27] Hsu Y Serpell JA. Development and validation of a questionnaire for measuring behavior and temperament traits dogs. J Am Vet Med Assoc. (2003) 223:1293–300. 10.2460/javma.2003.223.1293 14621216

[28] Konok V Dóka A Miklósi Á The behavior of the domestic dog (canis familiaris) during separation from and reunion with the owner: a questionnaire and an experimental study. Appl Anim Behav Sci. (2011) 135:300–8. 10.1016/j.applanim.2011.10.011

[29] Mills DS Dube MB Zulch H Stress and Pheromonatherapy in Small Animal Clinical Behaviour. Chichester: John Wiley & Sons (2013). 284 p.

[30] Palestrini C Minero M Cannas S Rossi E Frank D Video analysis of dogs with separation-related behaviors. Appl Anim Behav Sci. (2010) 124:61–7. 10.1016/j.applanim.2010.01.014

[31] Schäfer M. Nomothetic and idiographic methodology in psychiatry–a historical-philosophical analysis. Med Health Care Philos. (1999) 2:265–74. 10.1023/A:1009973828786 11080993

[32] Overall KL Clinical Behavioral Medicine for Small Animals. St. Louis, MO: Mosby-Year Book, Inc. (1997).

[33] Kendler KS Parnas J Philosophical Issues in Psychiatry IV: Psychiatric Nosology. Oxford: Oxford University Press (2017).

[34] Sheppard G Mills DS. Construct models in veterinary behavioural medicine: lessons from the human experience. Vet Res Commun. (2003) 27:175–91. 10.1023/A:1023372822712 12777092

[35] Mills DS Ewbank R ISAE, ethology and the veterinary profession. In: Brown JA Seddon YM Appleby MC, editors. Animals and us – 50 Years and More of Applied Ethology. Wageningen: Wageningen Academic Publishers (2016). p. 95–111.

[36] King JN Simpson BS Overall KL Appleby D Pageat P Ross C. Treatment of separation anxiety in dogs with clomipramine: results from a prospective, randomized, double-blind, placebo-controlled, parallel-group, multicenter clinical trial. Appl Animal Behav Sci. (2000) 67:255–75. 10.1016/S0168-1591(99)00127-6 10760607

[37] Simpson BS Landsberg GM Reisner IR Ciribassi JJ Horwitz D Houpt KA. Effects of reconcile (fluoxetine) chewable tablets plus behavior management for canine separation anxiety. Vet Ther. (2007) 8:18–31. 17447222

[38] Takeuchi Y Houpt KA Scarlett JM. Evaluation of treatments for separation anxiety in dogs. J Am Vet Med Assoc. (2000) 217:342–5. 10.2460/javma.2000.217.342 10935036

[39] Blackwell E Casey RA Bradshaw JWS Controlled trial of behavioural therapy for SRD in dogs. Vet Rec. (2006) 158:551–4. 10.1136/vr.158.16.551 16632528

[40] Sherman BL. Separation anxiety in dogs. Compend Contin Educ Vet. (2008) 30:27–42. 18278745

[41] Ainsworth Mary S. Infant-mother attachment. Am Psychol. (1979) 34:932. 517843 10.1037//0003-066x.34.10.932

[42] Mills DS Perspectives on assessing the emotional behavior of animals with behavior problems. Curr Opin Behav Sci. (2017) 16:66–72. 10.1016/j.cobeha.2017.04.002

[43] McCrave E. Diagnostic criteria for separation anxiety in the dog. Vet Clin North Am Small Anim Pract. (1991) 21:247–55. 10.1016/S0195-5616(91)50030-9 2053248

[44] Mislevy RJ Estimation of latent group effects. J Am Statist Assoc. (1985) 80:993–7. 10.1080/01621459.1985.10478215

[45] De Leeuw J Least squares optimal scaling of partially observed linear systems. In: van Montfort K Oud J Satorra A, editors. Recent Developments on Structural Equation Models. Dordrecht: Springer (2004). p. 121–34.

[46] Team RC R Development Core Team. R: A Language and Environment for Statistical Computing. Vienna: R Foundation for Statistical Computing (2018).

[47] Revelle WR psych: Procedures for Personality and Psychological Research. CRAN R Project. Evanston, IL: Northwestern University (2017).

[48] Bernaards C Jennrich R Package “GPArotation”. CRAN – R project (2015). Available online at: http://www.stat (accessed July 15, 2019).

[49] Kaiser HF The application of electronic computers to factor analysis. Educ Psychol Meas. (1960) 20:141–51. 10.1177/001316446002000116

[50] Cattell RB. The scree test for the number of factors. Multivariate Behav Res. (1966) 1:245–76. 10.1207/s15327906mbr0102_10 26828106

[51] Stevens J Applied Multivariate Statistics for the Social Sciences. Mahwah, NJ: Routledge (2002).

[52] Tabachnick BG Fidell LS Using Multivariate Statistics. 6th ed. Boston, MA: Pearson (2015). 1055 p.

[53] Maechler M cluster: “Finding Groups in Data”: Cluster Analysis Extended Rousseeuw et al. Hoboken, NJ: Wiley-interscience (2015).

[54] Moore WC Meyers DA Wenzel SE Teague WG Li H Li X. Identification of asthma phenotypes using cluster analysis in the severe asthma research program. Am J Respir Crit Care Med. (2010) 181:315–23. 10.1164/rccm.200906-0896OC 19892860 PMC2822971

[55] Weatherall M Travers J Shirtcliffe PM Marsh SE Williams MV Nowitz MR. Distinct clinical phenotypes of airways disease defined by cluster analysis. Eur Respir J. (2009) 34:812–8. 10.1183/09031936.00174408 19357143

[56] Jarek S mvnormtest: Normality Test for Multivariate Variables. CRAN R project (2012). Available online at: https://cran.r-project.org/web/packages/mvnormtest/mvnormtest.pdf (accessed July 21, 2019).

[57] R Core Team The R Package Stats (2018).

[58] Ogle D Ogle MD Package ‘FSA'. CRAN Repos. 1–206. (2017). (accessed July 15, 2019).

[59] Zarr JH Biostatistical Analysis. Upper Saddle River, NJ: Pearson Prentice-Hall (2010). 944 p.

[60] Klaus B Strimmer K. Package ‘fdrtool'. CRAN. (2015). Available online at: https://cran.r-project.org/web/packages/fdrtool/fdrtool.pdf (accessed on January 26, 2018).

[61] Hastie T Tibshirani R Package “mda”. CRAN R Project (2017). Available online at: https://cran.r-project.org/web/packages/mda/mda.pdf (accessed February 22, 2019).

[62] Kline P Psychometrics and Psychology. London, UK: Academic Press (1979).

[63] Field A Miles J Field Z Discovering Statistics Using R. London, UK: Sage Publications (2012). 958 p.

[64] Panksepp J Affective Neuroscience: The Foundations of Human and Animal Emotions. New York, NY: Oxford University Press (1998). 466 p.

[65] Podberscek L Hsu Y Serpell JA. Evaluation of clomipramine as an adjunct to behavioural therapy in the treatment of separation-related problems in dogs. Vet Rec. (1999) 145:365–9. 10.1136/vr.145.13.365 10573193

[66] Overall KL Dunham AE Frank D Frequency of nonspecific clinical signs in dogs with separation anxiety, thunderstorm phobia, and noise phobia, alone or in combination. J Am Vet Med Assoc. (2001) 219:467–73. 10.2460/javma.2001.219.467 11518172

[67] Ash P. The reliability of psychiatric diagnoses. J Abnorm Soc Psychol. (1949) 44:272. 10.1037/h0058417 18126462

[68] Silverman WK Saavedra LM Pina AA. Test-retest reliability of anxiety symptoms and diagnoses with the anxiety disorders interview schedule for DSM-IV: child and parent versions. J Am Acad Child Adolesc Psychiatr. (2001) 40:937–44. 10.1097/00004583-200108000-00016 11501694

[69] Landsberg GM Melese P Sherman BL Neilson JC Zimmerman A Clarke TP Effectiveness of fluoxetine chewable tablets in the treatment of canine separation anxiety. J Vet Behav Clin Appl Res. (2008) 3:12–9. 10.1016/j.jveb.2007.09.001

[70] Schwartz S. Separation anxiety syndrome in dogs and cats. J Am Vet Med Assoc. (2003) 222:1526–32. 10.2460/javma.2003.222.1526 12784957

[71] McPeake KJ Collins LM Zulch H Mills DS. The canine frustration questionnaire—development of a new psychometric tool for measuring frustration in domestic dogs (*Canis familiaris*). Front Vet Sci. (2019) 6:152. 10.3389/fvets.2019.00152 31165075 PMC6535675

[72] Zulch H Mills D Life Skills for Puppies. Dorchester: Veloce Publishing Ltd (2012). 91 p.

[73] Pageat P. Pathologie du Comportement du Chien. 2nd ed. Dorchester, UK: Maisond-Alfort: Le Point Veterinaire (1995). 380 p.

[74] Parthasarathy V Crowell-Davis SL Relationship between attachment to owners and separation anxiety in pet dogs (*Canis lupus familiaris*). J Vet Behav Clin Appl Res. (2006) 1:109–20. 10.1016/j.jveb.2006.09.005

[75] Sherman BL Mills DS. Canine anxieties and phobias: an update on separation anxiety and noise aversions. Vet Clin N Am Small Anim Pract. (2008) 38:1081–106. 10.1016/j.cvsm.2008.04.012 18672155

[76] Ainsworth MDS Bell SM Attachment, exploration, and separation: illustrated by the behaviour of one-year-olds in a strange situation. Child Dev. (1970) 41:49–67. 10.2307/1127388 5490680

[77] Panksepp J Clarici A Vandekerckhove M Yovell Y Neuro-evolutionary foundations of infant minds: from psychoanalytic visions of how primal emotions guide constructions of human minds toward affective neuroscientific understanding of emotions and their disorders. Psychoanal Inq. (2019) 39:36–51. 10.1080/07351690.2019.1549910

[78] Hall CS Emotional behavior in the rat. I. Defecation and urination as measures of individual differences in emot. J Comp Psychol. (1934) 18:385 10.1037/h0071444

[79] Goicoa A Fidalgo LE Suarez ML Espino L Perez-Lopez M Santamarina G. Zinc poisoning associated with separation anxiety in an Argentinean bulldog. Vet Hum Toxicol. (2002) 44:14–6. 11824765

[80] Lem M. Behavior moditication and pharmacotherapy for separation anxiety in a 2yo pointer cross. Can Vet J. (2002) 43:220. 11901597 PMC339210

[81] Elam M Thorén P Svensson TH. Locus coeruleus neurons and sympathetic nerves: activation by visceral afferents. Brain Res. (1986) 375:117–25. 10.1016/0006-8993(86)90964-9 3719350

[82] Kemeny ME The psychobiology of stress. Curr Dir Psychol Sci. (2003) 12:124–9. 10.1111/1467-8721.01246

[83] Main M Solomon J Procedures for identifying infants as disorganised/disoriented during the Ainsworth Strange Situation. In: Greenberg MT Cicchetti D Cummings EM, editors. Attachment in the Preschool Years. Chicago, IL: University of Chicago Press (1990). p. 121–60.

[84] Jones AC Gosling SD Temperament and personality in dogs (*Canis familiaris*): a review and evaluation of past research. Appl Anim Behav Sci. (2005) 95:1–53. 10.1016/j.applanim.2005.04.008

[85] Solomon J Beetz A Schöberl I Gee N Kotrschal K. Attachment security in companion dogs: adaptation of Ainsworth's strange situation and classification procedures to dogs and their human caregivers. Attach Hum Dev. (2019) 21:389–417. 10.1080/14616734.2018.1517812 30246604 PMC6532729

[86] Young DW Logical necessity and sufficiency in medicine. Math Biosci. (1974) 21:241–50. 10.1016/0025-5564(74)90018-2

[87] Sullivan FM MacNaughton RJ. Evidence in consultations : interpreted and individualised. Lancet. (1996) 348:941–3. 10.1016/S0140-6736(96)05219-1 8843817

