# Supplementary material for: Developing Diagnostic Frameworks in Veterinary Behavioral Medicine: Disambiguating Separation Related Problems in Dogs
Source: Front Vet Sci. 2020 Jan 17;6:499. doi: 10.3389/fvets.2019.00499 (PMC6978995; doi:10.3389/fvets.2019.00499)
Supplement: Supplementary file 1 [file Data_Sheet_1.docx]

Supplementary material

**Original online questionnaire about Separation related problems: A closer look at separation related problems in the dog**

**Introduction**

This study is being conducted as collaboration between the University of Lincoln, UK and the University of Veterinary Medicine and Pharmacy in Kosice, Slovakia.

This questionnaire is designed to improve our understanding of the dog’s behaviour in the owner’s absence. Destructiveness, vocalisation and house soiling are among the most common complaints of dog owners, frequently resulting in the breakdown of the human-animal bond and, in some cases, even leading to surrender and abandonment. By taking the time to fill out this questionnaire you will be helping to improve our knowledge on this important subject which may help us develop more effective treatments.

This survey is a translation of “A closer look at separation related problems”. If you have already completed the English version please do not fill in this one.

Please complete this questionnaire if your dog shows **ANY** of the following when separated from the owner or left alone:

- Depression / sadness
- Destructiveness
- Vocalisation: whining, barking or howling
- House soiling

Please **DO NOT** complete this survey if:

- You have owned your dog for less than 1 month
- Your dog is under 12 Weeks
- There has been a significant change in the household in the last month (e.g. family member left home, moved house etc)
- Your dog previously showed any of these signs and no longer does so

Please complete this questionnaire with a particular dog in mind and please complete only one survey per household, even if more than one dog shows the signs. 

The survey should take no longer than 20 to 25 minutes to complete and is completely anonymous. 

Thank you for taking the time to participate in this survey.

*Note: Where the questionnaire says “You”, we mean the individual to whom your dog seems most attached.

If you have any questions about the survey, please contact us at rmatos@lincoln.ac.uk**Owner’s information**

**Tell us a bit about yourself**

Female

Male

1. Owner´s gender
2. Your age
3. Country of residence
4. Is this the first dog that you’ve owned? Yes No
5. How many people live in the household?
6. How many dogs live in the household?
7. How many cats live in the household?
8. How many other free roaming animals live in the household (e.g. house rabbits)

**Dog’s information**

1. Dog’s age (in months or years)

in months

in years

🞎 Female

🞎 Male

1. Dog’s Gender:
2. Has this dog been spayed or neutered:

Yes 🞎 No 🞎

1. Dog’s breed (if mixed, select "mixed breed/unknown"):

This list of breeds is based on the breeds recognised by the Kennel Club (UK) and American Kennel Club. If you cannot find your dog's breed on the list please select "Other" and write it down on the space bellow.

1. Approximate weight:

In kg

In lbs

1. Where did you acquire this dog:

Bred him/her myself

From a breeder

From a shelter or rescue group

From a neighbor, friend or relative

From a pet-shop

Adopted as a stray

Other

Select an option

1. At what age did you acquire this dog?

**Note:** If you selected "bred him/her myself" in the previous question, please enter "0" (zero).

Bred him/her myself

Age in weeks

Age in months

Age in years

1. Where does the dog tend to spend the day?

At home

In the garden

Taken to work

Put in kennels

Other

Please select only one option

1. Is this dog currently suffering from any significant health problems? Yes 🞎 No 🞎
2. Please describe briefly:
3. Is your dog undergoing any medication? Yes 🞎 No 🞎
4. Please describe briefly:

**Main signs**

- Which of the following behaviours does your dog show in your absence?

|  |  |  |  | **Yes** | **No** | **Don’t know** |  |
| --- | --- | --- | --- | --- | --- | --- | --- |
|  | | Home soiling - urine |  |  | 🞎 | 🞎 | 🞎 |
|  | | House soiling - faeces |  |  | 🞎 | 🞎 | 🞎 |
|  | | Destructiveness |  |  | 🞎 | 🞎 | 🞎 |
|  | | Vocalisation |  |  | 🞎 | 🞎 | 🞎 |
|  | | Other |  |  | 🞎 | 🞎 | 🞎 |

1. Have these signs changed at any time since they started?

Yes No

1. if so please briefly describe at what age and any associated events in the space below
2. How seriously do you rate this as a problem 0=no problem at all 10 = very serious problem making me think about getting rid of the dog.

0

- What impact has this problem had on each of the following aspects of your quality of life?

|  |  |  | |  |  |  |  |  |
| --- | --- | --- | --- | --- | --- | --- | --- | --- |
|  | Your daily routine | | None  Minor  Moderate  Large  Not sure  Not applicable | |  |  |  |  |
|  | Your social life | | |  |  |  |  |  |
|  | Relationships with the people you live with | | |  |  |  |  |  |
|  | Relationships with family and friends you visit | | |  |  |  |  |  |
|  | Relationships with neighbours | |  | |  |  |  |  |
|  | Holidays/Weekend plans | |  | |  |  |  |  |
|  | Financial situation | |  | |  |  |  |  |

- Do any of the behaviours mentioned above (house soiling, vocalisation, destruction) improve if you leave your dog in any of the following circumstances?

|  |  |  |  | Yes  No  Don’t know  Not applicable |  |
| --- | --- | --- | --- | --- | --- |
|  | | With another human member of the household |  |  |  |
|  | | With another dog or cat |  |  |  |
|  | | With a person familiar to the dog (not part of the household) |  |  |  |
|  | | With a person unfamiliar to the animal |  |  |  |

1. After arriving home and finding one of the behaviours has occurred (dog vocalising, house soiled or destruction) how often do you (or other member of the household) verbally punish (scold, shout at, etc) your dog?

Never

Seldom

Sometimes

Usually

Always

1. How often do you (or other member of the household) verbally punish (scold, shout at, etc) your dog when he/she does other things you do not like or want?

Never

Seldom

Sometimes

Usually

Always

**Dogs may behave differently when left home alone compared to when they are not able to access their owner in the home. The next couple of questions compare your dog’s response in these two situations.**

- How often does your dog show each of the following behaviours when left home **alone** for **at least 1 hour**?
- Please indicate an option from **Never or Very rarely** (0 to 5% of time) to **Always or Nearly always** (96 to 100% of time) for each sign.

Never or Very rarely (0 to 5%)

Occasionally (6 to 35%)

Sometimes (36 to 65%)

Most times (66 to 95%)

Always or Nearly always (96 to 100%)

Don’t know

|  | |  |  |  |  |  |  |  |  |
| --- | --- | --- | --- | --- | --- | --- | --- | --- | --- |
|  | | House soiling | |  |  |  |  |  |  |
|  | | Destruction | |  |  |  |  |  |  |
|  | | Vocalization | |  |  |  |  |  |  |

- How often does your dog shown each of the following behaviours when **separated** from you within the home for **at least one hour**? (e.g. when in another room and without the possibility of accessing you)
- Please indicate an option from **Never or Very rarely** (0 to 5% of time) to **Always or Nearly always** (96 to 100% of time) for each sign.

Never or Very rarely (0 to 5%)

Occasionally (6 to 35%)

Sometimes (36 to 65%)

Most times (66 to 95%)

Always or Nearly always (96 to 100%)

Don’t know

|  | |  |  |  |  | |  | |  | |  | |  |  |
| --- | --- | --- | --- | --- | --- | --- | --- | --- | --- | --- | --- | --- | --- | --- |
|  | | House soiling | |  |  | |  | |  | |  | |  | |
|  | | Destruction | |  |  | |  | |  | |  | |  | |
|  | | Vocalization | |  |  | |  | |  | |  | |  | |

**Onset and changes**

⮚ When did each of the following behaviours start occurring?

Always been there or been there since the first month of ownership

Months or years after adoption

Not Applicable

|  |  |  | |  |  | | |  |
| --- | --- | --- | --- | --- | --- | --- | --- | --- |
|  | | House soiling |  |  | | |  |  |
|  | | Destruction |  |  |  |  |  |  |
|  | | Vocalization |  |  |  |  |  |  |

⮚ Have any of the following happened since acquiring this pet and was there a change in your dog’s behaviour around this time in relation to how he/she coped with being left alone at home (without human company) or separated from you or other family member (e.g. confined in another room) – tick all that apply

|  |  | Event occurred | Change in dog’s behaviour |  |
| --- | --- | --- | --- | --- |
|  | | Death of a family member | 🞎 | 🞎 |
|  | | Death of a pet | 🞎 | 🞎 |
|  | | Divorce | 🞎 | 🞎 |
|  | | Marriage | 🞎 | 🞎 |
|  | | Baby born | 🞎 | 🞎 |
|  | | Child moved | 🞎 | 🞎 |
|  | | Pet added | 🞎 | 🞎 |
|  | | Family moved | 🞎 | 🞎 |
|  | | Family schedule changed (lost or changed employment) | 🞎 | 🞎 |
|  | | Changed place where the dog spends most of its time | 🞎 | 🞎 |
|  | | Dog sick and in need of constant care | 🞎 | 🞎 |
|  | | Other | 🞎 | 🞎 |

**Separation periods**

⮚ How often is your dog left at home without human company for each of the following time-frames:

|  |  | Never  More rarely  1 – 2 times per week  3 – 6 times per week  Every day |  |  |  |  |  |
| --- | --- | --- | --- | --- | --- | --- | --- |
|  | | More than 8 hours |  | | | | |
|  | | 4 to 8 hours |  |  |  |  |  |
|  | | 1 to 4 hours |  |  |  |  |  |
|  | | Less than 1 hour |  |  |  |  |  |

1. During a week what is the longest time your dog is left?

Please fill in only one option

In minutes

In hours

1. How often in a week does this occur? (How many times)
2. **During the day**, when you need to leave your dog do you usually leave him/her

Please select one option

At home, free to roam through several rooms

At home restricted to a single room

At home confined in a crate

At home, in the garden free to roam

At home on a long line

At kennels

At friends or other familiar place

Other

1. **At night**, when you need to leave your dog do you usually leave him/her

Please select one option (idem previous question)

1. Do you take specific measures to reduce the risk of leaving your dog unsupervised when you need to go out?

Yes 🞎 No 🞎

- In order to reduce my concerns about my dog being left alone, I make the following changes to my life-style:

Tick all that apply.

|  |  |  | |  | |  |  |
| --- | --- | --- | --- | --- | --- | --- | --- |
|  | | Reduce how often I go out | |  | | 🞎 | |
|  | | Make sure someone in the household is always at home with the dog | |  | | 🞎 | |
|  | | Leave my dog at a dog hotel or day-care | |  | | 🞎 | |
|  | | Leave my dog at a friend’s or family’s house | |  | | 🞎 | |
|  | | Ask friends, neighbours, employers, family, etc to stay with my dog | |  | | 🞎 | |
|  | | Leave my dog with another dog or pet in the home | |  | | 🞎 | |
|  | | Other | |  | | 🞎 | |

**Your dog’s activity**

- Where does your dog normally sleep?

Tick all that apply

|  |  |  |  |  |
| --- | --- | --- | --- | --- |
|  | | In or on your bed | 🞎 | |
|  | | On its own bed on your bedroom | 🞎 | |
|  | | In a crate in your bedroom | 🞎 | |
|  | | On its own bed in another room | 🞎 | |
|  | | In a crate in another room | 🞎 | |
|  | | On the floor / chair etc next to your bed | 🞎 | |
|  | | In another room, voluntarily, anywhere he/she wants | 🞎 | |
|  | | In another room because you close off your bedroom, anywhere he/she wants | 🞎 | |
|  | | Other (please specify) |  | |

1. Which of the following best describes what your dog does the **majority** of the time while you are at home?

Ignores you

Stays in another room

Wanders from room to room including the room you are in

Stays in the room you are in

Watches you the whole time/Follows you from room to room

Stays in physical contact with you

Please select only one option

1. Does your dog:

Never

Seldom

Sometimes

Usually

Always

|  |  |  |  |  |  |  |  |
| --- | --- | --- | --- | --- | --- | --- | --- |
|  | Express distress in anticipation of you trying to withdraw from his/her presence | |  |  |  |  |  |
|  | Display a particularly strong bond for one member of the household | |  |  |  |  |  |
|  | Tend to follow you (or other members of household) about the house, from room to room. | |  |  |  |  |  |
|  | Tend to sit close to, or in contact with, you (or others) when you are sitting down. | |  |  |  |  |  |
|  | Have a particular object which he/she likes to have with him/her when you are not around  If yes, please describe bellow | |  |  |  |  |  |
|  | Have a particular place (e.g. a chair, crate, your bed etc) that he/she is particularly fond of going to when you are not around  If yes please describe bellow | |  |  |  |  |  |

Please describe the object and/or place here:

**Departures**

Dogs may react differently in response to regular predictable departures compared to less regular ones. The following questions focus on your dog’s reaction in these differing situations.

1. What does your dog do when you are getting ready to leave the house to go to work or other **daily routine**?

|  |  | **Yes** | **No** |  |
| --- | --- | --- | --- | --- |
|  | | No reaction | 🞎 | 🞎 |
|  | | Watches you | 🞎 | 🞎 |
|  | | Paces | 🞎 | 🞎 |
|  | | Whines/whimpers | 🞎 | 🞎 |
|  | | Salivates | 🞎 | 🞎 |
|  | | Looks anxious or “depressed” | 🞎 | 🞎 |
|  | | Trembles | 🞎 | 🞎 |
|  | | Other | 🞎 | 🞎 |
|  | |  |  |  |

1. What does your dog do when you are getting ready to leave the house at an **unusual or unexpected** time?

|  |  | **Yes** | **No** |  |
| --- | --- | --- | --- | --- |
|  | | No reaction | 🞎 | 🞎 |
|  | | Watches you | 🞎 | 🞎 |
|  | | Paces | 🞎 | 🞎 |
|  | | Whines/whimpers | 🞎 | 🞎 |
|  | | Salivates | 🞎 | 🞎 |
|  | | Looks anxious or “depressed” | 🞎 | 🞎 |
|  | | Trembles | 🞎 | 🞎 |
|  | | Other | 🞎 | 🞎 |

1. What does your dog normally do immediately after you have **stepped outside** the house?

|  |  | **Yes** | **No** | **Don’t know** |  |
| --- | --- | --- | --- | --- | --- |
|  | | No reaction | 🞎 | 🞎 | 🞎 |
|  | | Looks out the window | 🞎 | 🞎 | 🞎 |
|  | | Bites/claws at door(s)/window(s)/crate | 🞎 | 🞎 | 🞎 |
|  | | Vocalizes (whines, barks, howls) | 🞎 | 🞎 | 🞎 |
|  | | Other | 🞎 | 🞎 | 🞎 |

**Separation**

1. What does your dog do if your dog is **separated** from you at home for a brief time e.g. to take a shower/ go to the toilet and no one else is at home?

|  |  | **Yes** | **No** |  |
| --- | --- | --- | --- | --- |
|  | | Goes in with you | 🞎 | 🞎 |
|  | | Tries to go in but you don’t allow | 🞎 | 🞎 |
|  | | Scratches/ Bites/claws at the door | 🞎 | 🞎 |
|  | | Vocalises (whines, barks, howls) | 🞎 | 🞎 |
|  | | Salivates | 🞎 | 🞎 |
|  | | Looks anxious | 🞎 | 🞎 |
|  | | Looks depressed | 🞎 | 🞎 |
|  | | Trembles | 🞎 | 🞎 |
|  | | Other | 🞎 | 🞎 |

**Greeting behaviour**

1. What does your dog typically do when you return home and he/she was left alone without human company? For each condition please select the description that best describes your dog’s response.
2. When left for less than 1 hour

Ignores you

Greets you in a controlled way (comes up to you and noses or licks you etc)

Greets very enthusiastically, e.g. Jumps on you and/or vocalises

Follows you around the house till you acknowledge him/her

Avoids you

1. When left for more than 4 hours

Ignores you

Greets you in a controlled way (comes up to you and noses or licks you etc)

Greets very enthusiastically, e.g. Jumps on you and/or vocalises

Follows you around the house till you acknowledge him/her

Avoids you

1. What does your dog do when you return if left confined (in a room or part of the house) for a short while? Please select the description that best describes your dog’s response.

If this is not applicable in your situation select

*Not applicable*

Ignores you

Greets you in a controlled way (comes up to you and noses or licks you etc)

Greets very enthusiastically, e.g. Jumps on you and/or vocalises

Follows you around the house till you acknowledge him/her

Avoids you

Not applicable

**Vocalisations**

- Does your dog **bark** when…

Please tick all that apply

|  |  |  |
| --- | --- | --- |
|  | Phone rings | 🞎 |
|  | Doorbell rings or someone knocks on the door | 🞎 |
|  | He/she is shut somewhere | 🞎 |
|  | Asking for food | 🞎 |
|  | You have visits | 🞎 |
|  | Sees people or other dogs while is outside | 🞎 |
|  | Hears outside noise | 🞎 |
|  | Wants to be petted | 🞎 |
|  | Other | 🞎 |

- Does your dog **whine or howl** when…

Please tick all that apply

|  |  |  |
| --- | --- | --- |
|  | Phone rings | 🞎 |
|  | Doorbell rings or someone knocks on the door | 🞎 |
|  | He/she is shut somewhere | 🞎 |
|  | Asking for food | 🞎 |
|  | You have visits | 🞎 |
|  | Sees people or other dogs while is outside | 🞎 |
|  | Hears outside noise | 🞎 |
|  | Wants to be petted | 🞎 |
|  | Other | 🞎 |

1. Does your dog vocalise while alone at home (without human company):

Yes

No

Don’t know

1. What vocalisations does your dog make while alone at home (without human company):

|  |  | **Never** | | **Sometimes** | **Always** | | |
| --- | --- | --- | --- | --- | --- | --- | --- |
|  | Barking | 🞎  🞎  🞎 | 🞎  🞎  🞎 | | |  | 🞎 |
|  | Whining |  |  |  |  |  | 🞎 |
|  | Howling |  |  |  |  |  | 🞎 |

**Dog’s toileting habits**

1. Where does your dog urinate? Please tick all that apply

|  |  |  |
| --- | --- | --- |
|  | Outside | 🞎 |
|  | Newspapers/ puppy pads etc | 🞎 |
|  | In inappropriate places in the home when alone or shut in somewhere | 🞎 |
|  | In inappropriate places even when not alone or shut in | 🞎 |
|  | When approached, handled or picked up | 🞎 |
|  | When the owner arrives home | 🞎 |
|  | Other | 🞎 |

1. Where does your dog defecate? Please tick all that apply

|  |  |  |
| --- | --- | --- |
|  | Outside | 🞎 |
|  | Newspapers | 🞎 |
|  | In inappropriate places when it is alone or shut in somewhere | 🞎 |
|  | In inappropriate places even when is not alone or shut in | 🞎 |
|  | Other | 🞎 |

If your dog has never had house soiling problems after 6 months old **OR** if your dog is under 6 months old, please check the box and you will be taken to the next section.

Not Applicable 🞎

1. Has your dog **eliminated** while alone in the house after 6 months old (except when sick)?

Yes 🞎 No 🞎

Urination

Defecation

Both

1. If so, what type of elimination has occurred?

Less than once/month

1 to 3 times/month

1 to 4 times/week

Almost everyday

At least once/day

1. How frequently has this happened?

Please select only one option

1. Is it always in the same location? Yes 🞎 No 🞎
2. Do you ever find urine which has run down a wall or a chair (or other furniture)?

Yes

No

Not applicable

Yes

No

Not applicable

1. Are the faeces usually disturbed/scattered?
2. How long do you think your dog can comfortably stay indoors without having to go to the toilet?

Up to 4 hours

4 to 8 hours

8 to 12 hours

12 to 16 hours

**Objects in the home**

1. Do you usually leave toys, chews, and /or treats available for your dog when he/she is left alone?

Yes 🞎 No 🞎

1. Do you usually leave the radio or tv on for your dog when he/she is left alone?

Yes 🞎 No 🞎

If your dog is under 6 months old check the box N/A and you will be taken to the next section

Not Applicable 🞎

1. Regarding your objects (e.g. clothes, shoes, remote control, towels, etc), does your dog ever

|  |  | **Yes** | **No** |
| --- | --- | --- | --- |
|  | Guard a specific object | 🞎 | 🞎 |
|  | Take objects without destroying them | 🞎 | 🞎 |
|  | Take objects and destroys them while you are present | 🞎 | 🞎 |
|  | Take objects and destroys them while confined in a room without access to you | 🞎 | 🞎 |
|  | Takes objects and destroys them while left home alone without human company | 🞎 | 🞎 |

1. Has your dog **destroyed anything it was not supposed to** while **alone** in the house after **6 months** old? Yes 🞎 No 🞎

1. What was the size of the items destroyed?

|  |  | **Yes** | **No** |
| --- | --- | --- | --- |
|  | Small items (e.g. pens, papers, etc) | 🞎 | 🞎 |
|  | Medium-sized items (e.g. pillows, etc.) | 🞎 | 🞎 |
|  | Furniture, windows, doors, doorframes, other exit points from house | 🞎 | 🞎 |
|  | Structural damage (e.g. holes in wall, torn up linoleum, etc.) | 🞎 | 🞎 |

1. Which of the following items has your dog destroyed?

|  |  | **Yes** | **No** |
| --- | --- | --- | --- |
|  | Sofa or chair | 🞎 | 🞎 |
|  | Carpets | 🞎 | 🞎 |
|  | Doors | 🞎 | 🞎 |
|  | windows | 🞎 | 🞎 |
|  | Remote control or cell phone | 🞎 | 🞎 |
|  | Clothing | 🞎 | 🞎 |
|  | Other | 🞎 | 🞎 |

- How has your dog destroyed them?

|  |  |  | **Yes** | **No** | **Don’t know** |
| --- | --- | --- | --- | --- | --- |
|  | Using his mouth (biting, chewing, ripping) |  | 🞎 | 🞎 | 🞎 |
|  | Using his claws (scratching or digging motion) |  | 🞎 | 🞎 | 🞎 |
|  |  |  |  |  |  |

1. If the destruction has occurred to a sofa or door please indicate on the pictures the exact place(s) – noting how the door is hinged, location of door handle and your preferred seat on the sofa

Otherwise check the box *Not applicable* and you will be taken to the next section.

Not Applicable 🞎

1. If the destruction has occurred to a sofa, please answer the following questions.

If not, then please check the box Not applicable and you will be taken to the next set of questions.

Not Applicable 🞎

1. Please indicate the exact place(s) according to the picture. If this has occurred several times please refer to the first episode where there was significant damage to the furniture.

   If your sofa has only one seat ignore regions “o”, “q”, “k” and “m”.

place "i" 🞎

place "j" 🞎

place "k" 🞎

place "l" 🞎

place "m" 🞎

place "n" 🞎

place "o" 🞎

place "p" 🞎

place "q" 🞎

place "r" 🞎


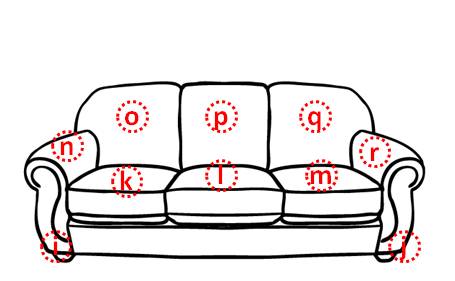


1

2

3

1. How many seats has your sofa?
2. In which place(s) did you usually sit before the destruction started (k, l or m)?

Place “k” 🞎

Place “l” 🞎

Place “m” 🞎

1. If the destruction has occurred to a doorway, please answer the following questions.

If not, then please check the box *Not applicable* and you will be taken to the next section.

Not Applicable 🞎

1. Please indicate the exact place(s) according to the picture. If this has occurred in several doorways please refer to where it most typically occurs.

a. (floor nearby the place where the door opens) 🞎

b. (floor nearby the place where the door is hinged) 🞎

c. (door frame next to where the door opens) 🞎

d. (door itself near where it opens) 🞎

e. (door itself in its bottom central area) 🞎

f. (door itself near where it is hinged) 🞎

g. (door frame next to where the door is hinged) 🞎

h. (on or around door handle) 🞎


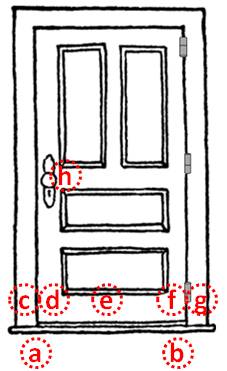


1. Was this the main exit from the room/space where the dog was left? Yes 🞎 No 🞎
2. Was the door closed at the time the damage occurred? Yes 🞎 No 🞎

**Reaction to noises**

1. Which of these describes most closely, what your dog does in each of the following situations. Please choose only one option

Stop what he/she was doing but quickly goes back to normal

Runs away and hides

Looks for me

Panics and starts to destroy things

Howls or whines

Barks

Don’t know

|  |  |  |
| --- | --- | --- |
|  |  | |
|  | Sudden loud noises (e.g. car backfire, gunshot, objects falling) | |
|  | Wind blowing | |
|  | Fireworks bangers | |
|  | Screeches / whistles | |
|  | Thunderstorms | |
|  | Road works, neighbours rearranging furniture, neighbours arguing loudly | |
|  | Garbage truck, vacuum cleaner, lawn mower | |

**Other behaviours**

1. Does your dog do any of the following, more frequently than you think is normal for a dog?

|  |  |  | | **Yes** | **No** |
| --- | --- | --- | --- | --- | --- |
|  | Licks / mouths his legs and/or feet | |  | 🞎 | 🞎 |
|  | Licks / mouths other part of the body | |  | 🞎 | 🞎 |
|  | Licks / mouths some place or object in the house (door, floor, walls) | |  | 🞎 | 🞎 |
|  | Licks / mouths a specific object (e.g. large stones | |  | 🞎 | 🞎 |
|  | Sucks its bedding | |  | 🞎 | 🞎 |
|  | Mouths /Licks the owner | |  | 🞎 | 🞎 |
|  | Other | |  | 🞎 | 🞎 |

1. How often does your dog show each of the following signs when **confined or left home alone**

Never

Seldom

Sometimes

Usually

Always

Don't know

|  |  |  |  |  |  |  |  |
| --- | --- | --- | --- | --- | --- | --- | --- |
|  | Loss of appetite |  |  |  |  |  |  |
|  | Excessive salivation |  |  |  |  |  |  |
|  | Withdrawal, inactivity, “depression” |  |  |  |  |  |  |
|  | Restlessness, agitation or pacing |  |  |  |  |  |  |

- Please indicate how often your dog does each of the following

Never

Sometimes

Always

1. Tries to lick your hands and face
2. Shows puppyish behaviour
3. Explores new things using his/her mouth
4. Enjoys chewing for long periods of time
5. When given the chance would play all the time
6. Tends to cower or make himself smaller when someone approaches
7. Shows high levels of activity
8. Has your dog has ever shown aggressive behaviour when you’re trying to leave the house

Yes 🞎 No 🞎

1. Has your dog has ever been aggressive to you in other contexts

Yes 🞎 No 🞎

**Dog’s behaviour in specific situations**

- Does your dog tend to roll on its back when

|  |  |  |  |  |  |
| --- | --- | --- | --- | --- | --- |
|  | Approached by an unfamiliar dog |  |  | Yes  No  Don’t know |  |
|  | Approached by an unfamiliar person |  |  |  |  |
|  | Looked in the eye |  |  |  |  |

- What is your dog’s reaction in the following situations? Please select only one option

**When he/she can´t have something that he /she can see in the following situations:**

1. You arrive home but meet a neighbour on your driveway and stay chatting for a few minutes. Meanwhile your dog can see you from the window.
2. Your dog is playing with a toy that accidentally goes under the furniture where he/she can’t reach it.

Barks and/or whines

Growls

Bites or tries to bite

Ignores me

It’s destructive (e.g. scratches at door)

Toilets

Wag’s his/her tail

Looks depressed

Backs away

Don’t know

Not applicable

**Entry to his/her territory / personal space**

1. Your dog sees an unfamiliar dog or person come near the home but can’t reach them due to some barrier (gate, lead, etc))
2. Your dog is in the car and an unfamiliar dog or person comes near
3. Someone unfamiliar to the dog approaches and tries to pet him/her on the head
4. Somebody comes to the door

Barks and/or whines

Growls

Bites or tries to bite

It’s destructive (e.g. scratches at door)

Toilets

Wag’s his/her tail

Looks depressed

Backs away

Don’t know

Not applicable

**When he/she gets less that it was expected**

1. you try to put him/her back on the lead earlier than normal after a run in the park or when he/she gets a significantly shorter walk than usual
2. When you substitute his/her favourite treats with different ones, which may not be as good as the original.

Barks and/or whines

Growls

Bites or tries to bite

Ignores me

It’s destructive (e.g. scratches at door)

Toilets

Wag’s his/her tail

Looks depressed

Backs away

Don’t know

Not applicable

1. If you try to take either a favourite food or toy from your dog,
   what sort of response might he/she show?

**When he/she doesn’t get what he/she was expecting**

1. He / she doesn’t get the normal special treat given after a walk or a similar disappointment
2. He is not allowed to play free in the park or do some other usual activity one day

Barks and/or whines

Growls

Bites or tries to bite

Ignores me

It’s destructive (e.g. scratches at door)

Toilets

Wag’s his/her tail

Looks depressed

Backs away

Don’t know

Not applicable

**Supplementary Figure 1. Bar plot of 157 variables of the Separation related problems questionnaire in 345 dogs showing the eigenvalue according to number of principal components.**

**Supplementary Table 1. Final result of the Principal Component Analysis of 56 behaviours from 345 dogs with separation related problems**. **Only items loading >0.4 are shown for clarity. Using this criterion there was no cross-loading between components.**

| Variables | PC1 | PC2 | PC3 | PC4 | PC5 | PC6 | PC7 |
| --- | --- | --- | --- | --- | --- | --- | --- |
| Destruction of the main exit door when it was closed | 0.96 |  |  |  |  |  |  |
| Destruction of the main exit door of the room | 0.93 |  |  |  |  |  |  |
| Destruction of door frame next to where the door opens | 0.92 |  |  |  |  |  |  |
| Destruction of door itself next to where it opens | 0.9 |  |  |  |  |  |  |
| Destruction of doors | 0.87 |  |  |  |  |  |  |
| Destruction on or around door handle | 0.74 |  |  |  |  |  |  |
| Destruction of big objects (furniture, windows, doors, doorframes, other exit points from house) | 0.67 |  |  |  |  |  |  |
| Destruction of floor nearby the place where the door opens | 0.61 |  |  |  |  |  |  |
| Destruction of house structure (holes in wall, torn up linoleum) | 0.57 |  |  |  |  |  |  |
| Destruction using his/her claws | 0.54 |  |  |  |  |  |  |
| Vocalization after owner has stepped outside |  | 0.76 |  |  |  |  |  |
| Whines during unusual pre departures |  | 0.67 |  |  |  |  |  |
| Frequency of vocalization when dog is left alone for at least 1h (often and always) |  | 0.66 |  |  |  |  |  |
| Whines during routinely pre departures |  | 0.66 |  |  |  |  |  |
| Frequency of vocalization when dog is left confined for at least 1h (often and always) |  | 0.62 |  |  |  |  |  |
| Paces during routinely pre departures |  | 0.61 |  |  |  |  |  |
| Frequency of distress pre departure (often and always) |  | 0.61 |  |  |  |  |  |
| Frequency of whining without human company (always) |  | 0.59 |  |  |  |  |  |
| Vocalizes without human company |  | 0.59 |  |  |  |  |  |
| Looks anxious during short separation period |  | 0.55 |  |  |  |  |  |
| Vocalizes during short separation period |  | 0.54 |  |  |  |  |  |
| Paces during unusual pre departures |  | 0.51 |  |  |  |  |  |
| Bites and/or claws the door/window/crate after owner has stepped outside |  | 0.49 |  |  |  |  |  |
| Frequency of restlessness, agitation or pacing when confined of left home alone (often and always) |  | 0.48 |  |  |  |  |  |
| Frequency of barking without human company (always) |  | 0.45 |  |  |  |  |  |
| Barks when confined |  | 0.45 |  |  |  |  |  |
| Urinates in owner absence |  |  | 0.82 |  |  |  |  |
| Urinates in inappropriate places when alone or confined |  |  | 0.8 |  |  |  |  |
| Urine when alone that started only after 6 months old |  |  | 0.79 |  |  |  |  |
| Defecates in owner absence |  |  | 0.78 |  |  |  |  |
| Defecates in inappropriate places when alone or confined |  |  | 0.77 |  |  |  |  |
| Feaces when alone that started only after 6 months old |  |  | 0.72 |  |  |  |  |
| Frequency of house soiling when left alone for at least 1h (often and always) |  |  | 0.65 |  |  |  |  |
| Frequency of house soiling when alone that happened only after 6 months old (often and always) |  |  | 0.63 |  |  |  |  |
| Urinates in inappropriate places even when not alone or confined |  |  | 0.45 |  |  |  |  |
| Defecates in inappropriate places even when not alone or confined |  |  | 0.44 |  |  |  |  |
| Takes objects and destroys them when alone without human company |  |  |  | 0.87 |  |  |  |
| Destruction using his/her mouth |  |  |  | 0.81 |  |  |  |
| Destruction of medium-sized items |  |  |  | 0.77 |  |  |  |
| Destruction of clothing |  |  |  | 0.71 |  |  |  |
| Destructiveness in owner absence |  |  |  | 0.65 |  |  |  |
| Take objects and destroy them when confined |  |  |  | 0.64 |  |  |  |
| Destruction of carpets |  |  |  | 0.46 |  |  |  |
| Barks when there's a person at the door |  |  |  |  | 0.87 |  |  |
| Wags tail when there's a person at the door |  |  |  |  | - 0.81 |  |  |
| Barks when doorbell rings |  |  |  |  | 0.75 |  |  |
| Wags tail when he/she is inside of the car and an unfamiliar person/dog approaches |  |  |  |  | - 0.66 |  |  |
| Barks when he/she is inside of the car and an unfamiliar person/dog approaches |  |  |  |  | 0.65 |  |  |
| Barks when it can't reach an unfamiliar person/dog when approaching |  |  |  |  | 0.57 |  |  |
| Growls when he/she sees the owner outside talking to some person |  |  |  |  |  | 0.99 |  |
| Bites when the owner tries to put him/her on the lead earlier than normal after a run in the park or when he/she gets a significantly shorter walk than usual. |  |  |  |  |  | 0.99 |  |
| Bites when he/she is not allowed to play free in the park or do some other usual activity one day |  |  |  |  |  | 0.99 |  |
| Panics and starts to destroy things when hears fireworks bangers |  |  |  |  |  |  | 0.89 |
| Panics and starts to destroy things when hears thunderstorms |  |  |  |  |  |  | 0.85 |
| Panics and starts to destroy things when hears screeches or whistles |  |  |  |  |  |  | 0.83 |
| Panics and starts to destroy things when hears sudden loud noises (e.g. car backfires, objects falling) |  |  |  |  |  |  | 0.82 |
| Proportion Explained (%) | 12 | 10 | 9 | 7 | 6 | 6 | 6 |

**Supplementary Figure 2 Bar plot showing eigenvalues of principal components of 56 variables of the total population 417 dogs.**

**Supplementary Table 2. Final result of the Principal Component Analysis of 56 behaviours from 417 dogs with separation related problems**. **Only items loading >0.3 are shown for clarity. Using this criterion there was no cross-loading between components.**

| Variables | PC1 | PC2 | PC3 | PC4 | PC5 | PC6 | PC7 |
| --- | --- | --- | --- | --- | --- | --- | --- |
| Destruction of the main exit door when it was closed | 0.95 |  |  |  |  |  |  |
| Destruction of the main exit door of the room | 0.91 |  |  |  |  |  |  |
| Destruction of door frame next to where the door opens | 0.87 |  |  |  |  |  |  |
| Destruction of door itself next to where it opens | 0.86 |  |  |  |  |  |  |
| Destruction of doors | 0.79 |  |  |  |  |  |  |
| Destruction of floor nearby the place where the door opens | 0.67 |  |  |  |  |  |  |
| Destruction on or around door handle | 0.61 |  |  |  |  |  |  |
| Destruction of big objects (furniture, windows, doors, doorframes, other exit points from house) | 0.54 |  |  |  |  |  |  |
| Destruction of house structure (holes in wall, torn up linoleum) | 0.49 |  |  |  |  |  |  |
| Destruction of carpets | 0.39 |  |  | 0.34 |  |  |  |
| Urinates in owner absence |  | 0.82 |  |  |  |  |  |
| Defecates in owner absence |  | 0.81 |  |  |  |  |  |
| Urinates in inappropriate places when alone or confined |  | 0.77 |  |  |  |  |  |
| Feaces when alone that started only after 6 months old |  | 0.77 |  |  |  |  |  |
| Defecates in inappropriate places when alone or confined |  | 0.77 |  |  |  |  |  |
| Urine when alone that started only after 6 months old |  | 0.76 |  |  |  |  |  |
| Frequency of house soiling when left alone for at least 1h (often and always) |  | 0.56 |  |  |  |  |  |
| Frequency of house soiling when alone that happened only after 6 months old (often and always) |  | 0.5 |  |  |  |  |  |
| Urinates in inappropriate places even when not alone or confined |  | 0.46 |  |  |  |  |  |
| Defecates in inappropriate places even when not alone or confined |  | 0.41 |  |  |  |  |  |
| Whines during routinely pre departures |  |  | 0.68 |  |  |  |  |
| Whines during unusual pre departures |  |  | 0.64 |  |  |  |  |
| Paces during routinely pre departures |  |  | 0.63 |  |  | 0.33 |  |
| Vocalization after owner has stepped outside |  |  | 0.62 |  |  |  |  |
| Frequency of vocalization when dog is left alone for at least 1h (often and always) |  |  | 0.62 |  |  |  |  |
| Paces during unusual pre departures |  |  | 0.58 |  |  | 0.35 |  |
| Frequency of whining without human company (always) |  |  | 0.57 |  |  |  |  |
| Vocalizes without human company |  |  | 0.55 |  |  |  |  |
| Frequency of restlessness, agitation or pacing when confined of left home alone (often and always) |  |  | 0.53 |  |  |  |  |
| Vocalizes during short separation period |  |  | 0.52 |  |  |  |  |
| Frequency of distress pre departure (often and always) |  |  | 0.48 |  |  |  |  |
| Bites and/or claws the door/window/crate after owner has stepped outside |  |  | 0.47 |  |  |  |  |
| Frequency of vocalization when dog is left confined for at least 1h (often and always) |  |  | 0.46 |  |  |  |  |
| Looks anxious during short separation period |  |  | 0.45 |  |  |  |  |
| Frequency of barking without human company (always) |  |  | 0.37 |  |  |  |  |
| Barks when confined |  |  |  |  |  |  |  |
| Takes objects and destroys them when alone without human company |  |  |  | 0.84 |  |  |  |
| Destruction using his/her mouth |  |  |  | 0.8 |  |  |  |
| Destruction of medium-sized items |  |  |  | 0.8 |  |  |  |
| Take objects and destroy them when confined |  |  |  | 0.74 |  |  |  |
| Destruction of clothing |  |  |  | 0.73 |  |  |  |
| Destructiveness in owner absence |  |  |  | 0.65 |  |  |  |
| Destruction using his/her claws | 0.4 |  |  | 0.46 |  |  |  |
| Barks when there's a person at the door |  |  |  |  | 0.85 |  |  |
| Wags tail when there's a person at the door |  |  |  |  | -0.81 |  |  |
| Barks when doorbell rings |  |  |  |  | 0.73 |  |  |
| Wags tail when he/she is inside of the car and an unfamiliar person/dog approaches |  |  |  |  | -0.63 |  |  |
| Barks when he/she is inside of the car and an unfamiliar person/dog approaches |  |  |  |  | 0.57 |  |  |
| Barks when it can't reach an unfamiliar person/dog when approaching |  |  |  |  | 0.57 |  |  |
| Panics and starts to destroy things when hears screeches or whistles |  |  |  |  |  | 0.71 |  |
| Panics and starts to destroy things when hears fireworks bangers |  |  |  |  |  | 0.69 |  |
| Panics and starts to destroy things when hears sudden loud noises (e.g. car backfires, objects falling) |  |  |  |  |  | 0.61 |  |
| Panics and starts to destroy things when hears thunderstorms |  |  |  |  |  | 0.34 |  |
| Bites when the owner tries to put him/her on the lead earlier than normal after a run in the park or when he/she gets a significantly shorter walk than usual. |  |  |  |  |  |  | 0.99 |
| Bites when he/she is not allowed to play free in the park or do some other usual activity one day |  |  |  |  |  |  | 0.99 |
| Proportion Explained (%) | 11 | 9 | 9 | 8 | 6 | 4 | 4 |

**No dog presented the behaviour “Growls when he/she sees the owner outside talking to some person” within this sample**.

**Supplementary Figure 3. Bar plot showing eigenvalues of principal components of 56 variables of the total population 762 dogs.**

**Supplementary Table 3 Final result of the series of Principal Component Analyses (PCA) performed initially on 762 dogs presenting with separation related problems regarding 56 items which were further reduced to 54 grouped across seven principal components. For each variable (item) its frequency and loadings in each PC are detailed. In addition, the total percent of variation explained for each PC is given and interpretative label specified in the last two rows.**

| Variables | *N (%)* | PC1 | PC2 | PC3 | PC4 | PC5 | PC6 | PC7 |
| --- | --- | --- | --- | --- | --- | --- | --- | --- |
| Destruction of the main exit door when it was closed | *121 (15.9)* | **0.95** | 0 | 0.01 | -0.01 | 0.01 | 0 | -0.03 |
| Destruction of the main exit door of the room | *112 (14.7)* | **0.91** | -0.01 | 0 | -0.02 | 0.03 | 0 | -0.06 |
| Destruction of door frame next to where the door opens | *93 (12.2)* | **0.89** | -0.02 | 0 | -0.04 | -0.02 | 0.01 | 0 |
| Destruction of door itself next to where it opens | *87 (11.4)* | **0.89** | -0.03 | 0.01 | -0.07 | 0.02 | 0.01 | -0.03 |
| Destruction of doors | *147 (19.3)* | **0.83** | 0.06 | 0.01 | 0.11 | 0 | -0.01 | 0.04 |
| Destruction on or around door handle | *52 (6.8)* | **0.68** | 0.01 | -0.04 | -0.05 | -0.07 | 0.02 | 0.04 |
| Destruction of floor nearby the place where the door opens | *50 (6.6)* | **0.64** | 0.07 | 0 | -0.03 | -0.01 | 0 | 0.01 |
| Destruction of big objects (furniture, windows, doors, doorframes, other exit points from house) | *197 (25.9)* | **0.6** | 0.06 | 0.01 | 0.29 | -0.03 | -0.03 | 0.07 |
| Destruction of house structure (holes in wall, torn up linoleum) | *110 (14.4)* | **0.54** | -0.01 | 0.04 | 0.21 | 0.04 | -0.02 | 0.13 |
| Destruction using his/her claws | *256 (33.6)* | **0.45** | 0.12 | -0.03 | 0.41 | 0.03 | -0.05 | 0.07 |
| Vocalization after owner has stepped outside | *514 (67.5)* | -0.03 | **0.7** | -0.02 | 0.02 | 0.08 | 0.01 | -0.09 |
| Whines during routinely pre-departures | *280 (36.8)* | -0.04 | **0.67** | -0.02 | -0.03 | -0.04 | -0.04 | 0.01 |
| Whines during unusual pre-departures | *319 (41.9)* | -0.03 | **0.66** | 0.02 | -0.03 | -0.01 | -0.05 | 0.01 |
| Frequency of vocalization when dog is left alone for at least 1h (often and always) | *444 (59.3)* | 0.04 | **0.65** | -0.01 | -0.05 | 0.08 | 0.03 | -0.14 |
| Paces during routinely pre-departures | *305 (40)* | 0.01 | **0.63** | -0.04 | -0.01 | -0.11 | -0.02 | 0.2 |
| Frequency of whining without human company (always) | *254 (33.3)* | 0.06 | **0.59** | 0 | -0.03 | -0.01 | -0.01 | -0.09 |
| Vocalizes without human company | *591 (77.6)* | 0.01 | **0.58** | -0.02 | -0.02 | 0.11 | 0.01 | -0.08 |
| Frequency of distress pre-departure (often and always) | *258 (33.9)* | 0.06 | **0.55** | -0.03 | -0.04 | -0.01 | -0.02 | 0.03 |
| Paces during unusual pre-departures | *351 (46.1)* | 0.01 | **0.55** | -0.02 | -0.01 | -0.03 | 0.03 | 0.19 |
| Frequency of vocalization when dog is left confined for at least 1h (often and always) | *287 (37.7)* | -0.01 | **0.54** | 0.02 | -0.02 | 0.02 | 0.07 | -0.08 |
| Vocalizes during short separation period | *264 (34.7)* | -0.09 | **0.53** | 0.04 | 0.1 | 0.01 | 0.07 | -0.02 |
| Frequency of restlessness, agitation or pacing when confined of left home alone (often and always) | *254 (33.3)* | 0.07 | **0.51** | 0.06 | 0.08 | -0.1 | -0.02 | 0.12 |
| Looks anxious during short separation period | *209 (27.4)* | -0.06 | **0.5** | 0.01 | 0.09 | -0.05 | 0.09 | 0.04 |
| Bites and/or claws the door/window/crate after owner has stepped outside | *231 (30.3)* | 0.18 | **0.48** | 0.11 | 0.1 | -0.05 | -0.07 | -0.02 |
| Frequency of barking without human company (always) | *243 (31.9)* | 0.11 | **0.41** | 0.04 | -0.04 | 0.2 | 0.07 | -0.13 |
| Urinates in owner absence | *213 (27.9)* | 0.02 | 0.01 | **0.81** | -0.04 | -0.01 | -0.02 | -0.01 |
| Defecates in owner absence | *150 (19.7)* | -0.01 | 0 | **0.8** | -0.01 | -0.02 | -0.01 | -0.04 |
| Urinates in inappropriate places when alone or confined | *163 (21.4)* | -0.07 | 0.03 | **0.78** | 0.03 | 0.01 | -0.02 | 0.05 |
| Defecates in inappropriate places when alone or confined | *125 (16.4)* | -0.02 | -0.03 | **0.77** | 0.04 | -0.02 | -0.01 | 0.03 |
| Urine when alone that started only after 6 months old | *238 (31.2)* | 0.01 | -0.01 | **0.77** | 0 | 0.02 | -0.03 | 0.01 |
| Faeces when alone that started only after 6 months old | *192 (25.2)* | 0.03 | -0.02 | **0.75** | -0.01 | 0.05 | -0.03 | -0.01 |
| Frequency of house soiling when left alone for at least 1h (often and always) | *69 (9.1)* | 0.04 | 0.07 | **0.6** | 0.02 | -0.09 | 0.07 | 0.01 |
| Frequency of house soiling when alone that happened only after 6 months old (often and always) | *50 (6.6)* | 0.13 | -0.08 | **0.56** | -0.06 | 0 | 0.1 | -0.02 |
| Urinates in inappropriate places even when not alone or confined | *66 (8.7)* | -0.08 | -0.02 | **0.46** | 0.04 | 0.04 | 0.12 | -0.06 |
| Defecates in inappropriate places even when not alone or confined | *43 (5.6)* | -0.01 | 0.02 | **0.44** | -0.02 | 0.05 | 0.16 | -0.05 |
| Takes objects and destroys them when alone without human company | *323 (42.4)* | -0.02 | -0.04 | 0.01 | **0.86** | 0.02 | 0 | -0.04 |
| Destruction using his/her mouth | *441 (57.9)* | 0.11 | -0.08 | -0.05 | **0.82** | 0.03 | -0.01 | 0.01 |
| Destruction of medium-sized items | *278 (36.5)* | -0.01 | 0.01 | -0.02 | **0.8** | -0.07 | 0.01 | 0.01 |
| Destruction of clothing | *214 (28.1)* | -0.09 | 0.01 | 0.04 | **0.74** | 0.04 | 0.01 | -0.09 |
| Take objects and destroy them when confined without human company | *197 (25.9* | -0.11 | 0.11 | 0.08 | **0.7** | 0.04 | 0.02 | 0.02 |
| Destructiveness in owner absence | *346 (45.4)* | 0.16 | 0.01 | -0.02 | **0.64** | -0.08 | 0.06 | 0.05 |
| Barks when there's a person at the door | *574 (75.3)* | 0.01 | -0.03 | -0.02 | 0 | **0.86** | -0.01 | -0.02 |
| Wags tail when there's a person at the door | *148 (19.4)* | 0.02 | -0.05 | 0 | -0.03 | **-0.82** | 0.02 | -0.03 |
| Barks when doorbell rings | *582 (76.4)* | 0.04 | -0.01 | 0.03 | -0.09 | **0.74** | -0.01 | 0.06 |
| Wags tail when he/she is inside of the car and an unfamiliar person/dog approaches | *182 (23.9)* | 0.09 | -0.06 | -0.01 | -0.07 | **-0.64** | 0 | -0.08 |
| Barks when he/she is inside of the car and an unfamiliar person/dog approaches | *388 (50.9)* | -0.01 | 0 | 0 | 0.07 | **0.6** | 0.04 | -0.01 |
| Barks when it can't reach an unfamiliar person/dog when approaching | *482 (63.3)* | 0.08 | -0.04 | -0.03 | -0.03 | **0.58** | 0.03 | -0.02 |
| Bites when the owner tries to put him/her on the lead earlier than normal after a run in the park or when he/she gets a significantly shorter walk than usual. | *2 (0.3)* | 0 | 0.02 | -0.02 | -0.01 | 0 | **0.97** | 0.01 |
| Bites when he/she is not allowed to play free in the park or do some other usual activity one day | *2 (0.3)* | 0 | 0.02 | -0.02 | -0.01 | 0 | **0.97** | 0.01 |
| Growls when he/she sees the owner outside talking to some person | *1 (0.1)* | 0 | -0.04 | 0.05 | 0.04 | 0 | **0.85** | 0 |
| Panics and starts to destroy things when hears screeches or whistles | *3 (0.4)* | -0.06 | 0.01 | -0.01 | -0.01 | 0.04 | 0.01 | **0.81** |
| Panics and starts to destroy things when hears fireworks bangers | *10 (1.3)* | 0.01 | -0.06 | 0.02 | -0.03 | 0.02 | 0.01 | **0.79** |
| Panics and starts to destroy things when hears thunderstorms | *6 (0.8)* | 0.02 | -0.01 | 0 | -0.06 | 0.04 | 0.01 | **0.74** |
| Panics and starts to destroy things when hears sudden loud noises (e.g. car backfires, objects falling) | *5 (0.7)* | 0.01 | 0.03 | -0.01 | 0.03 | -0.03 | 0.01 | **0.7** |
| *% variance explained* |  | ***11*** | ***9*** | ***9*** | ***8*** | ***6*** | ***5*** | ***5*** |
| Interpretative label |  | **Exit frustration** | **Social Panic** | **Elimination** | **Redirected frustration** | **Reactive communication** | **Immediate frustration** | **Noise sensitivity** |

**Bold items load >0.4 and were retained as features of the principal component.**

**Supplementary Table 4. Number and percentage of 133 dogs presenting with separation related problems (group A) assigned to each sub-group according to the type of cluster analysis. HACA: hierarchical agglomerative; and CAPAM: partitioning around medoids.**

| HACA | A.1 (42) | % | A.2 (47) | % | A.3 (44) | % |
| --- | --- | --- | --- | --- | --- | --- |
| CAPAM |  |  |  |  |  |  |
| E.1 (33) | 3 | 7.1 | 5 | 10.6 | ***25*** | 56.8 |
| % | 9.1 |  | 15.2 |  | 75.8 |  |
| E.2 (44) | ***36*** | 85.7 | 1 | 2.1 | 7 | 15.9 |
| % | 81.8 |  | 2.3 |  | 15.9 |  |
| E.3 (56) | 3 | 7.1 | ***41*** | 87.2 | 12 | 27.3 |
| % | 5.4 |  | 73.2 |  | 21.4 |  |

**Bold and italic numbers indicate to which group most dogs were assigned. Underlined numbers on the right of the bold and italic ones are related to the Hierarchical agglomerative cluster analysis (HACA) while those under the bold and italic are related to the Cluster analysis using partitioning around medoids method (CAPAM).**

**Supplementary Table 5. Number and percentage of 221 dogs presenting with separation related problems (group B) assigned to each sub-group according to the type of cluster analysis. HACA: hierarchical agglomerative; and CAPAM: partitioning around medoids.**

| HACA | B.1 (103) | % | B.2 (78) | % | B.3 (40) | % |
| --- | --- | --- | --- | --- | --- | --- |
| CAPAM |  |  |  |  |  |  |
| F.1 (52) | 21 | 20.4 | 24 | 30.8 | 7 | 17.5 |
| % | 40.4 |  | 46.2 |  | 13.5 |  |
| F.2 (96) | ***40*** | 38.8 | ***53*** | 68 | 3 | 7.5 |
| % | 41.7 |  | 55.2 |  | 3.1 |  |
| F.3 (73) | ***42*** | 40.8 | 1 | 1.3 | ***30*** | 75 |
| % | 57.5 |  | 1.4 |  | 41.1 |  |

**Bold and italic numbers indicate to which group most dogs were assigned. Underlined numbers on the right of the bold and italic ones are related to the Hierarchical agglomerative cluster analysis (HACA) while those under the bold and italic are related to the Cluster analysis using partitioning around medoids method (CAPAM).**

**Supplementary Table 6. Number and percentage of 271 dogs presenting with separation related problems (group C) assigned to each sub-group according to the type of cluster analysis. HACA: hierarchical agglomerative; and CAPAM: partitioning around medoids.**

| HACA | C1 (128) | % | C2 (27) | % | C3 (116) | % |
| --- | --- | --- | --- | --- | --- | --- |
| CAPAM |  |  |  |  |  |  |
| G1 (86) | ***86*** | 67.2 | 0 | 0 | 0 | 0 |
| % | 100 |  | 0 |  | 0 |  |
| G2 (73) | 16 | 12.5 | ***27*** | 100 | 30 | 25.9 |
| % | 21.9 |  | 37 |  | 41.1 |  |
| G3 (112) | 26 | 20.3 | 0 | 0 | ***86*** | 74.1 |
| % | 23.2 |  | O |  | 76.8 |  |

**Bold and italic numbers indicate to which group most dogs were assigned. Underlined numbers on the right of the bold and italic ones are related to the Hierarchical agglomerative cluster analysis (HACA) while those under the bold and italic are related to the Cluster analysis using partitioning around medoids method (CAPAM).**

**Supplementary Table 7.** **Number and percentage of 137 dogs presenting with separation related problems (group D) assigned to each sub-group according to the type of cluster analysis. HACA: hierarchical agglomerative; and CAPAM: partitioning around medoids.**

| HACA | D.1 (88) | % | D.2 (49) | % |
| --- | --- | --- | --- | --- |
| CAPAM |  |  |  |  |
| H.1 (86) | ***84*** | 95.5 | 2 | 4.1 |
| % | 97.7 |  | 2.3 |  |
| H.2 (51) | 4 | 4.6 | ***47*** | 95.9 |
| % | 7.8 |  | 92.2 |  |

**Bold and italic numbers indicate to which group most dogs were assigned. Underlined numbers on the right of the bold and italic ones are related to the Hierarchical agglomerative cluster analysis (HACA) while those under the bold and italic are related to the Cluster analysis using partitioning around medoids method (CAPAM).**
